# Radiochemical and Biological Evaluation of 3p-*C*-NETA-ePSMA-16, a Promising PSMA-Targeting Agent for Radiotheranostics

**DOI:** 10.3390/ph16060882

**Published:** 2023-06-15

**Authors:** Erika Murce, Stephen Ahenkorah, Savanne Beekman, Maryana Handula, Debra Stuurman, Corrina de Ridder, Frederik Cleeren, Yann Seimbille

**Affiliations:** 1 Department of Radiology and Nuclear Medicine, University Medical Center Rotterdam, Erasmus MC, 3015 GD Rotterdam, The Netherlands; e.murcesilva@erasmusmc.nl (E.M.); s.beekman@erasmusmc.nl (S.B.); m.handula@erasmusmc.nl (M.H.); d.stuurman@erasmusmc.nl (D.S.); c.deridder@erasmusmc.nl (C.d.R.); 2 Erasmus MC Cancer Institute, 3015 GD Rotterdam, The Netherlands; 3 NURA Research Group, Belgian Nuclear Research Center (SCK CEN), 2400 Mol, Belgium; stephen.ahenkorah@kuleuven.be; 4 Radiopharmaceutical Research, Department of Pharmacy and Pharmacology, University of Leuven, 3000 Leuven, Belgium; frederik.cleeren@kuleuven.be; 5 TRIUMF, Life Sciences Division, Vancouver, BC V6T 2A3, Canada

**Keywords:** 3p-*C*-NETA, bifunctional chelator, prostate cancer, PSMA, theranostics

## Abstract

Bifunctional chelators (BFCs) are a key element in the design of radiopharmaceuticals. By selecting a BFC that efficiently complexes diagnostic and therapeutic radionuclides, a theranostic pair possessing almost similar biodistribution and pharmacokinetic properties can be developed. We have previously reported 3p-*C*-NETA as a promising theranostic BFC, and the encouraging preclinical outcomes obtained with [^18^F]AlF-3p-*C*-NETA-TATE led us to conjugate this chelator to a PSMA-targeting vector for imaging and treatment of prostate cancer. In this study, we synthesized 3p-*C*-NETA-ePSMA-16 and radiolabeled it with different diagnostic (^111^In, ^18^F) and therapeutic (^177^Lu, ^213^Bi) radionuclides. 3p-*C*-NETA-ePSMA-16 showed high affinity to PSMA (IC_50_ = 4.61 ± 1.33 nM), and [^111^In]In-3p-*C*-NETA-ePSMA-16 showed specific cell uptake (1.41 ± 0.20% ID/10^6^ cells) in PSMA expressing LS174T cells. Specific tumor uptake of [^111^In]In-3p-*C*-NETA-ePSMA-16 was observed up to 4 h p.i. (1.62 ± 0.55% ID/g at 1 h p.i.; 0.89 ± 0.58% ID/g at 4 h p.i.) in LS174T tumor-bearing mice. Only a faint signal could be seen at 1 h p.i. in the SPECT/CT scans, whereas dynamic PET/CT scans performed after administration of [^18^F]AlF-3p-*C*-NETA-ePSMA-16 in PC3-Pip tumor xenografted mice resulted in a better tumor visualization and imaging contrast. Therapy studies with short-lived radionuclides such as ^213^Bi could further elucidate the therapeutic potential of 3p-*C*-NETA-ePSMA-16 as a radiotheranostic.

## 1. Introduction

Radioligand therapy (RLT) of cancers has been developing at a fast pace in recent years, with the clinical translation of promising radiopharmaceuticals such as [^177^Lu]Lu-PSMA-617 [1,2] (Pluvicto^TM^, lutetium Lu 177 vipivotide tetraxetan) for treatment of metastatic castrate-resistant prostate cancer, and [^177^Lu]Lu-DOTA-TATE [3] (Lutathera^®^) for treatment of neuroendocrine tumors. Companion diagnosis and personalized dosimetry have further cemented RLT as a promising strategy in personalized medicine [4]. Within this, theranostic (diagnostic + therapeutic) agents are of great interest, as the same precursor can be used for both diagnostic screening and monitoring of treatment and for the therapy itself. Such an approach improves the accuracy of personalized dosimetry estimations for RLT, as the radiotracers have almost identical pharmacokinetics [5]. Different radionuclides are available and can be selected based on their particular physical properties. Ideally, the physical half-life of the radionuclide should match the biological half-life of the biovector used.

The radionuclide chelator plays an important role in the complexation of the therapeutic or diagnostic radionuclide. Bifunctional chelators (BFCs) are able to complex a radiometal and also possess a modification that allows them to be attached to a targeting vector. They should form stable complexes which do not suffer dissociation or transchelation by other metals in the body [6,7]. Ideally, radiolabeling should also be possible at mild temperatures to avoid degradation of biomolecules and with fast kinetics, which is of particular importance for short-lived radionuclides. Several BFCs have been developed throughout the years to efficiently capture a broad range of radiometals, which differ in their physico-chemical properties (such as ligand donor atom preferences, coordination geometry, number, electronegativity, and oxidation state) [8].

One of the most commonly used chelators is 2,2′,2″,2′′′-(1,4,7,10-tetraazacyclododecane-1,4,7,10-tetrayl)tetraacetic acid (DOTA), a macrocyclic chelator considered as a ‘gold standard’ due to its ability to stably form complexes with a wide range of radiometals (such as ^111^In, ^177^Lu, ^86/90^Y, ^225^Ac, and ^44/47^Sc) [7]. However, the downside of macrocyclic chelators is their slow kinetics, often requiring high temperatures and long incubation times for metal complexation, which can be detrimental when labeling heat-sensitive biomolecules or short-lived radiometals. Acyclic chelators, such as 2,2′,2″,2′′′-{[(carboxymethyl)azanediyl]bis(ethane-2,1-diylnitrilo)}tetraacetic acid (DTPA), offer fast kinetics and allow for radiolabeling at milder temperatures, but present the drawback of forming less stable complexes.

In order to harness the benefits of both classes of chelators, ligands combining both a macrocycle connected to acyclic arms have been developed, such as 2-(4,7-biscarboxymethyl[1,4,7]triazacyclo nona-1-yl-ethyl)car-bonyl-methylamino]acetic acid (NETA). The macrocyclic ring of NETA is based on the structure of 2,2′,2”-(1,4,7-triazacyclononane-1,4,7-triyl)triacetic acid (NOTA), with the replacement of one carboxylate group by a bis(carboxymethyl)amino donor group connected to the macrocycle by an ethylene moiety [9]. Other chelators from this class, containing both a macrocycle and acyclic moieties such as NEPA [10], NET3A [11], and DEPA [12], have been reported to stably complex a variety of different radionuclides.

Derivatization of NETA generates BFCs such as 3p-*C*-NETA, 3p-*C*-NETA-NCS [13], or 3p-*C*-NETA-(*t*Bu)_4_-oxa-butanoic acid in a few synthetic steps [14]. 3p-C-NETA radiolabeled with ^88^Y, ^177^Lu, and ^205/206^Bi was shown to be highly stable in serum in vitro for up to 11 days. In particular, the ^86^Y and ^177^Lu complexes showed high stability and rapid clearance from the body, with low organ uptake [9]. Conjugation of the chelator 3p-C-NETA-NCS with the antibody trastuzumab led to the evaluation of ^90^Y-, ^177^Lu- and ^205/206^Bi-3p-*C*-NETA-trastuzumab, all showing a good biodistribution profile when evaluated in vivo [13,15]. 3p-*C*-NETA has also recently been shown to form stable radiocomplexes with ^161^Tb [16] and ^213^Bi [15]. Finally, the positron-emitting radionuclides ^68^Ga and ^18^F (in the form of Al^18^F) were also evaluated in combination with 3p-*C*-NETA. Despite efficient complexation of ^68^Ga, the radiocomplex dissociated in serum. [^18^F]AlF-3p-*C*-NETA, however, showed excellent complex stability up to 4 h in both PBS and serum [14]. The versatility of 3p-*C*-NETA, indicated by its ability to stably complex a broad range of radionuclides, illustrates its potential as a true theranostic chelator.

Despite the extensive evaluation of 3p-*C*-NETA, including preliminary evaluation after conjugation with trastuzumab for radioimmunotherapy [15], further studies in conjugation with smaller targeting vectors have been limited. Recently, we conjugated 3p-*C*-NETA to an SSTR2 (somatostatin receptor 2) targeting TATE ((Tyr^3^)-octreotate) vector and synthesized [^18^F]AlF-3p-*C*-NETA-TATE in good radiochemical yield (RCY) and high radiochemical purity (RCP). [^18^F]AlF-3p-*C*-NETA-TATE showed fast blood clearance and specific uptake in SSTR2-expressing organs, as well as low uptake to other organs, when evaluated in healthy female Wistar rats [14]. Such results not only warrant further studies in tumor-bearing mice but also show the potential of 3p-*C*-NETA as a chelator for small-molecule and peptide-based radiopharmaceuticals.

**Figure 1:**
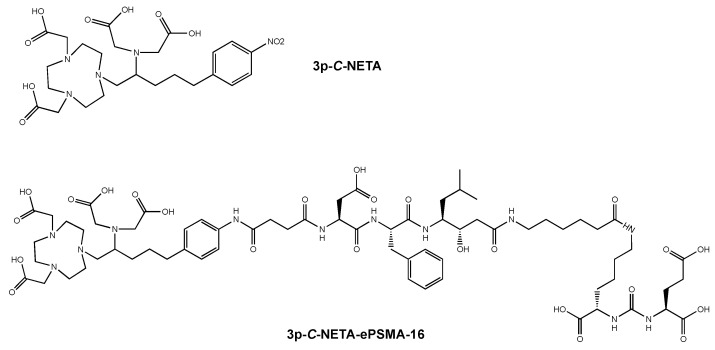
Structure of the chelator 3p-*C*-NETA and the compound 3p-*C*-NETA-ePSMA-16.

This prompted us to further investigate this chelator in combination with other therapeutic targets, starting with the design of a prostate-specific membrane antigen (PSMA) targeting radiopharmaceutical. Here, we present the chemical synthesis of 3p-*C*-NETA-ePSMA-16 (Figure 1) and its radiolabeling with the diagnostic radionuclides ^111^In (SPECT) and Al^18^F (PET) and therapeutic radionuclides ^177^Lu (β-emitter) and ^213^Bi (α- and β-emitter). The binding affinity of 3p-*C*-NETA-ePSMA-16 was evaluated via an enzymatic assay, and [^111^In]In-3p-*C*-NETA-ePSMA-16 was further evaluated on LS174T PSMA+ cells for cell uptake and internalization. [^111^In]In-3p-*C*-NETA-ePSMA-16 was then evaluated in BALB/c nu/nu mice transfected with LS174T PSMA+ tumor xenografts for ex vivo biodistribution and in vivo biodistribution with SPECT/CT imaging. Finally, to further validate the versatility of this probe, we evaluated [^18^F]AlF-3p-*C*-NETA-ePSMA-16 in SCID mice bearing both PSMA^+^ and PSMA^−^ tumor xenografts using dynamic PET/CT imaging.

## 2. Results

The PSMA-targeting moiety **1** was synthesized by Fmoc-based solid phase peptide synthesis (SPPS), whereas conjugation of the chelator and deprotection of the protecting groups were accomplished in liquid phase. This approach was selected since we observed some degradation of the compound during the acidic cleavage from the resin when the chelator was conjugated to **1** in solid phase. 3p-*C*-NETA-ePSMA-16 was obtained in high chemical purity (>95%) after purification, as verified by LC-MS (Figure S1). The overall chemical yield was approximately 10%, but no attempt to optimize or scale up the synthetic procedure was carried out at this stage. 3p-*C*-NETA-ePSMA-16 was stable for >1 year when stored as a lyophilized powder and >6 months when stored as an aqueous solution. Titration of the compound allowed us to determine the exact peptide content in the solution, which is essential for the radiochemical and biological tests.

Radiolabeling with ^111^In afforded quantitative radiochemical yield (RCY) (99.7 ± 0.2%, *n* = 5) in mild conditions (rt, 5 min) and radiochemical purity (RCP) >99% (Table 1). The compound was stable in PBS and mouse serum for 24 h (RCP > 95%, Figures S2 and S3) and also showed good stability during the transchelation challenge (93.2 ± 0.7% intact [^111^In]In-3p-*C*-NETA-ePSMA-16 at 24 h, *n* = 3, Figure S4). Radiolabeling with precursor amounts as low as 0.05 nmol afforded RCY > 95% (10 MBq/0.05 nmol, A_m_ = 200 MBq/nmol, Figure S5). Radiolabeling of 3p-*C*-NETA-ePSMA-16 with ^177^Lu in similar conditions gave low RCYs (RCY < 40% at 50 MBq/1 nmol, rt, 20 min; *n* = 2). However, high RCYs (93.6 ± 2.0; *n* = 3) were obtained when the reaction mixture was heated at 90 °C for 5 min. [^177^Lu]Lu-3p-*C*-NETA-ePSMA-16 remained stable (RCP > 89%) in PBS and mouse serum up to 24 h post-incubation (Figures S6 and S7). [^213^Bi]Bi-3p-*C*-NETA-ePSMA-16 was obtained with excellent RCC (94%, *n* = 1). [^213^Bi]Bi-3p-*C*-NETA-ePSMA-16 was stable in human serum for up to 4 h after incubation (97.4 ± 2.4%; *n* = 3, Figure S8). [^18^F]AlF-3p-*C*-NETA-ePSMA-16 was obtained with good RCC (40%, *n = 1*) and 97.4% RCP (A_m_ = 22 GBq/µmol). The compound remained stable in mouse serum for at least 4 h (96.1 ± 0.8%; *n* = 3, Figure S9).

**Figure 2:**
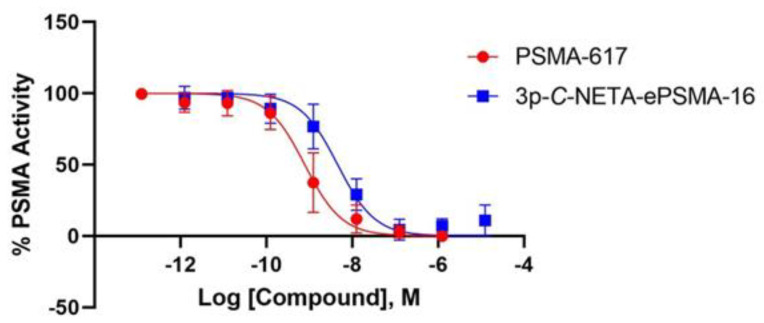
Representative curve for the IC_50_ determination for 3p-*C*-NETA-ePSMA-16 and PSMA-617 via NAALADase enzymatic assay. The percentage of enzymatic activity is plotted against the log of the concentrations. PSMA-617 is used as an internal reference for assay reproducibility. The results show the average and standard deviation of three separate assays performed in triplicate.

**Figure 3:**
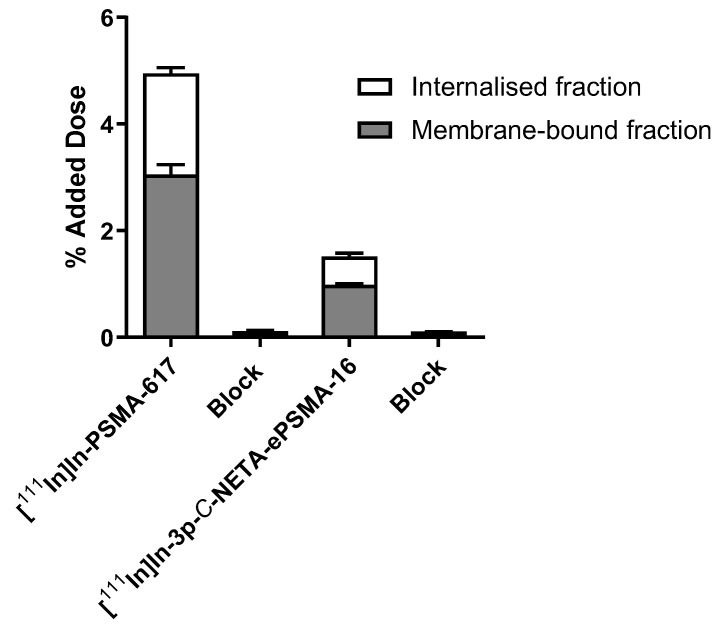
Cell uptake and internalization of [^111^In]In-PSMA-617 and [^111^In]In-3p-*C*-NETA-ePSMA-16 in LS174T PSMA+ cells. Values are expressed as a percentage of added dose, and the experiment was performed in triplicate.

3p-*C*-NETA-ePSMA-16 showed a good affinity to PSMA with an IC_50_ value 5-fold higher than the IC_50_ value found for the reference PSMA-617 (4.61 ± 1.33 nM vs. 0.78 ± 0.30 nM) (Figure 2). [^111^In]In-3p-*C*-NETA-ePSMA-16 also retained high hydrophilicity, with a logD value of −2.95 ± 0.19 (*n* = 3) (logD of PSMA-617 = −2.00) [1]. The uptake and internalization assay in PSMA-transfected LS174T cells demonstrated that two third of the uptake of [^111^In]In-3p-*C*-NETA-ePSMA-16 was located on the membrane (0.90 ± 0.05% AD/10^6^ cells) and one-third of the tracer was internalized (0.50 ± 0.16% AD/10^6^ cells, Figure 3; *n* = 3). [^111^In]In-PSMA-617 also showed a similar ratio between internalized and membrane-bound uptake (membrane-bound fraction: 2.95 ± 0.55% AD/10^6^ cells; internalized fraction: 1.88 ± 0.30% AD/10^6^ cells). The higher overall uptake of [^111^In]In-PSMA-617 was expected, considering it has a better binding affinity to PSMA.

**Figure 4:**
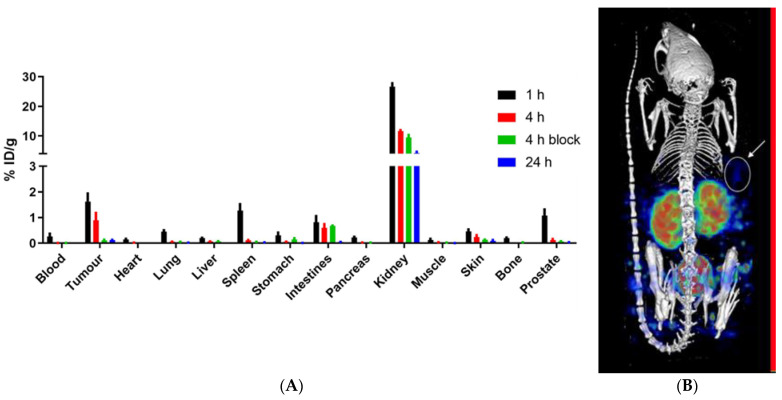
Biodistribution of [^111^In]In-3p-*C*-NETA-ePSMA-16 in BALB/c nude mice bearing LS174T tumor xenografts. (**A**) Ex vivo biodistribution. Organ and tissue uptake is represented as a percentage of injected dose per gram. The collection was performed at 1, 4, and 24 h p.i., with a block group also at 4 h. (**B**) Representative MIP images of SPECT/CT scans at 1 h p.i. (right flank; *n* = 4; tumor indicated by white arrow). The color scale represents % ID/mL (minimum = 0.003% ID/mL, maximum = 0.2% ID/mL).

Biodistribution studies with [^111^In]In-3p-*C*-NETA-ePSMA-16 were carried out in PSMA+ LS174T tumor-bearing BALB/c mice (Figure 4A). [^111^In]In-3p-*C*-NETA-ePSMA-16 showed a good tumor uptake at early time points and was cleared from the tumor over time (1.62 ± 0.55% ID/g, 0.89 ± 0.58% ID/g and 0.12 ± 0.02% ID/g at 1, 4 and 24 h p.i., respectively). PSMA-specificity was verified by co-injection of a 50-fold excess of unlabeled 3p-*C*-NETA-ePSMA-16. A significantly (*p* = 0.03) lower tumor uptake was observed in the blocked group at 4 h p.i. (0.12 ± 0.04% ID/g compared to 4 h: 0.89 ± 0.58% ID/g). High kidney uptake was observed at all time points, with the highest uptake at 1 h p.i. (26.70 ± 2.12% ID/g). Significantly lower uptake at 4 h p.i. (11.62 ± 0.62% ID/g, *p* < 0.0001) and 24 h p.i. (4.27 ± 0.87% ID/g) suggests a fast renal clearance of the compound. This uptake is partially PSMA-mediated, as it was reduced in the block group (9.53 ± 1.66% ID/g, compared to 11.62 ± 0.62% ID/g at 4 h p.i., *p* = 0.006). The spleen (1.28 ± 0.49% ID/g) and prostate (1.08 ± 0.41% ID/g) also showed uptake at 1 h p.i.; however, radiation was quickly cleared from these organs. The intestines also showed uptake up to 4 h p.i., which was not PSMA-specific, as it was also observed in the blocked group. Blood uptake (0.26 ± 0.19% ID/g at 1 h p.i.) was notably low even at the earliest time point, suggesting that the compound is rapidly cleared out from the body. All the results of the ex vivo biodistribution can be found in Table S1.

SPECT/CT imaging at 1 h p.i. (Figure 4B) showed high uptake in the kidneys and in the bladder, highlighting the rapid excretion of the compound from the body. Only a faint signal was observed in the tumor. At 24 h p.i., only the kidneys were visible, with the radioactivity in the tumor and other organs already cleared, which is in accordance with the data obtained from the ex vivo biodistribution.

**Figure 5:**
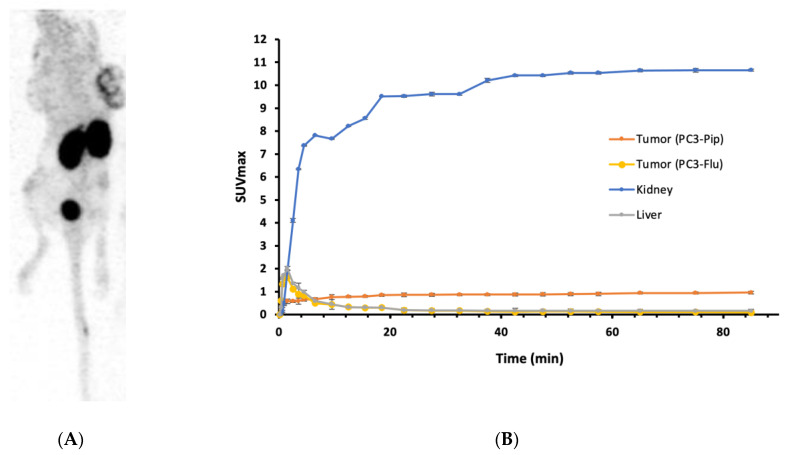
PET/CT imaging of SCID mice bearing PC3-Pip (right flank) and PC3-flu (left flank) tumor xenografts after administration of [^18^F]AlF-3p-*C*-NETA-ePSMA-16. (**A**) 75-85 min MIP image of a PET/CT scan. (**B**) SUV_max_ values obtained for tumor (PC3-Pip (PSMA^+^) and PC3-Flu (PSMA^−^)), kidneys, and liver during dynamic PET scans up to 85 min p.i. (right flank; *n* = 4).

Dynamic PET scans of [^18^F]AlF-3p-*C*-NETA-ePSMA-16 were acquired up to 85 min post-injection. PC3-Pip (PSMA^+^) tumor uptake was rapidly observed (SUV = 0.58 ± 0.09 at 1.5 min) and slowly increased until reaching a plateau throughout the whole duration of the scans (SUV = 0.95 ± 0.05 at 85 min). In contrast, low uptake was observed in the PC3-Flu tumor (SUV = 0.09 ± 0.04 at 85 min), indicating PSMA specific tumor accumulation of [^18^F]AlF-3p-*C*-NETA-ePSMA-16. Liver uptake peaked at 2 min p.i. (SUV = 2.02 ± 0.07) but was rapidly cleared out, with SUV < 0.2 after 20 min. Kidney uptake rapidly increased during the first 3 min, eventually reaching a plateau of SUV ≈ 10. The radioactivity was not cleared from the kidneys during the duration of the experiment (up to 85 min p.i.). At 75–85 min, the MIP images showed a strong signal in the kidneys and bladder, with a moderate PC3-Pip tumor uptake and low PC3-Flu tumor uptake. (Figure 5). Uptake in other organs was not observed. The full table of SUV values in the tumor, kidneys, and liver can be found in Table S2.

## 3. Discussion

The bifunctional chelator (BFC) is one of the key components of a radiopharmaceutical, as it connects the targeting vector and the radionuclide and is also responsible for keeping the radiometal stably complexed. Poorly complexed radionuclides dissociate from the chelator and, therefore, from the radiopharmaceutical in vivo, leading to accumulation in healthy organs. This, in consequence, results in low-quality images, poor target visualization, and high radiation dose to these organs, depending on whether the radionuclide is used for diagnosis or therapy. Therefore, careful evaluation of the complex stability is necessary when developing new radiopharmaceuticals or attempting new chelator-radiometal combinations [7]. Moreover, advances in RLT, with the investigation of new α- and β-emitters, call for developing and evaluating novel suitable chelators [17].

Chong et al. have developed and extensively evaluated the NETA chelator, both in its non-functionalized form and as a BFC [11,13,18,19]. We recently reported the synthesis of 3p-*C*-NETA-TATE, where 3p-*C*-NETA was conjugated to an SSTR2 targeting vector. [^18^F]AlF-3p-*C*-NETA-TATE was obtained in good RCY (41.4 ± 8%, *n* = 8, apparent molar activity 22 ± 8 GBq/μmol, >97% RCP) and showed a good biodistribution profile with rapid blood clearance in healthy Wistar rats. The only other example in the literature of conjugation of the functionalized 3p-*C*-NETA to a small biovector was reported by Kang et al. [18].

Aiming to investigate the potential of 3p-*C*-NETA as a chelator in small-molecule-based radiopharmaceuticals, we synthesized a PSMA targeting ligand containing 3p-*C*-NETA. A short hydrophilic linker was attached to the EuK binding moiety to improve the binding to PSMA. Then, the bifunctional chelator was conjugated to obtain our final product, 3p-*C*-NETA-ePSMA-16. We validated the affinity of 3p-*C*-NETA-ePSMA-16 towards PSMA by performing an enzymatic binding affinity assay. The compound exhibited a good affinity to PSMA (IC_50_ = 4.61 ± 1.33 nM), despite the linker and chelator modifications. Chelators can influence not only the binding of radiopharmaceuticals but also the pharmacokinetics of the compound due to charge and lipophilicity changes [20]. Uptake and internalization were evaluated in LS174T PSMA+ cells using the indium-labeled PSMA targeting ligand with or without a 50-fold excess of the unlabeled compound to determine specificity. [^111^In]In-3p-*C*-NETA-ePSMA-16 had a total uptake of 1.41 ± 0.20% ID/10^6^ cells, with no uptake observed in the blocked group. While this was lower than the total uptake of [^111^In]In-PSMA-617 (4.84 ± 0.83% AD/10^6^ cells), this difference is expected considering that the binding affinity of 3p-*C*-NETA-ePSMA-16 was 5-fold lower than that of PSMA-617.

^111^In (t_1/2_  =  2.8 d, Eγ = 171 and 245 keV, EC 100%) has not been previously evaluated with 3p-*C*-NETA. 3p-*C*-NETA-ePSMA-16 demonstrated complexation of ^111^In quantitatively at room temperature within 5 min, even at low precursor amounts (0.05 nmol). We performed additional transchelation studies to verify the stability of the radio complex, which was >90% stable in excess of EDTA up to 24 h post-incubation. [^111^In]In-3p-*C*-NETA-ePSMA-16 remained intact up to 24 h post-incubation in PBS and mouse serum at 37 °C, further illustrating the stability of this radiocomplex.

Introduction of the Al^18^F-radiolabeling strategy [21,22] as a facile, one-pot method to stably complex ^18^F (t_1/2_  =  109.7 min, Eβ^+^ 635 keV, 97%) gave rise to a wide range of new radiopharmaceuticals, but also to the search of suitable chelators to complex Al^18^F. Because of the smaller coordination sphere of Al^3+^, larger chelators such as DOTA are not efficient in complexing Al^18^F, whereas 1,4,7-triazacyclononane-*N*,*N*′,′-diacetic acid (NODA), which has a smaller cavity, has been shown to stably chelate this complex [22]. While good RCCs (30–50%) can be obtained from NODA complexation reactions, heating is required. NODA is also not compatible with therapeutic radiometals with larger coordination spheres, such as ^177^Lu, ^213^Bi or ^225^Ac. The cyclic moiety of 3p-*C*-NETA is derived from the macrocycle NOTA, and 3p-*C*-NETA was shown to efficiently chelate Al^18^F [14]. Here, we obtained [^18^F]AlF-3p-*C*-NETA-ePSMA-16 in good RCC (40%) and excellent RCP (97.4%), and the complex remained intact (>95%) in human serum up to 4 h post-incubation.

Envisioning future therapeutic studies with 3p-*C*-NETA-ePSMA-16, we also performed a preliminary evaluation of [^177^Lu]Lu-3p-*C*-NETA-ePSMA-16 and [^213^Bi]Bi-3p-*C*-NETA-ePSMA-16. 3p-*C*-NETA has been shown to instantly bind to ^177^Lu (t_1/2_ = 6.65 d, Eβ^−^ = 134 keV), retaining high complex stability in serum. It was also more efficient in chelating ^177^Lu compared to the decadentate 3p-*C*-NEPA, which also contains macrocyclic and acyclic donor groups. 2-[(carboxymethyl)][5-(4-nitrophenyl-1-[4,7,10-tris(carboxymethyl)-1,4,7,10-tetraazacyclododecan-1-yl]pentan-2-yl)amino]acetic acid) (3p-*C*-DEPA), another decadentate hybrid chelator derived from the DOTA macrocycle [23] was also less suitable for the complexation of ^177^Lu, with [^177^Lu]Lu-3p-*C*-DEPA showing a loss of 25% stability in human serum [10]. 3p-*C*-NETA, therefore, seems to be the best-suited candidate in this class of chelators for the complexation of ^177^Lu. However, we were not able to obtain [^177^Lu]Lu-3p-*C*-NETA-ePSMA-16 in good RCY at room temperature, and heating at 90 °C for at least 5 min was required for full incorporation of the radionuclide. It is likely due to either the presence of the PSMA-targeting moiety or the difference in the atomic radius of Lu^+3^ and In^+3^. [^177^Lu]Lu-3p-*C*-NETA has, however, been previously obtained with an RCC > 99% when labeling was performed at 25 °C for 10–12 min [13,14]. When incubated with PBS and mouse serum, [^177^Lu]Lu-3p-*C*-NETA-ePSMA-16 remained highly stable for 24 h (89% intact product in PBS and mouse serum).

The short half-life of ^213^Bi (t_1/2_ = 45.6 min, Eα = 8.4 MeV, γ = 440 keV) makes it an interesting radionuclide for targeted alpha therapy with small molecules and peptides, which are rapidly excreted from the body. ^213^Bi is available from a ^225^Ac/^213^Bi generator, making it accessible to institutions without a cyclotron. ^205/6^Bi-3p-*C*-NETA-trastuzumab showed high in vitro and in vivo stability, with a good biodistribution profile and high tumor uptake. Tumor uptake increased over time (10.80 ± 2.2% ID/g at 2 h; 24.10 ± 4.8% ID/g at 24 h), while blood uptake decreased but remained elevated, in accordance with the long blood half-life of antibodies (21.89 ± 5.9% ID/g at 2 h; 9.92 ± 2.2% ID/g at 24 h). Excretion organs, such as kidneys and liver, peaked at 6 h p.i., but decreased at 24 h [15]. Considering the short half-life of ^212/3^Bi, evaluating ^212/3^Bi-3p-*C*-NETA with short-lived biological vectors is crucial to elucidate whether this chelator is suitable for this radionuclide. We obtained [^213^Bi]Bi-3p-C-NETA-ePSMA-16 in a good RCC (94%) after labeling with short reaction times, illustrating how promising 3p-*C*-NETA is as a chelator considering the short half-life of ^213^Bi. The complex remained stable in human serum up to 4 h post-incubation, suggesting that 3p-*C*-NETA is a good chelator for Bismuth.

We performed imaging and biodistribution studies with 3p-C-NETA-ePSMA-16 radiolabeled with the diagnostic radionuclides ^111^In and Al^18^F to determine if this probe is suitable for imaging PSMA-positive tumors. To do so, we used both PET/CT and SPECT/CT imaging, and we employed two different PSMA-expressing tumor cell lines, LS174T and PC3-PIP, as well as a PSMA-negative cell line, PC3-flu. These cell lines, which are extensively used in the literature, allow for a comparison of our results with other studies. Biodistribution studies with [^111^In]In-3p-C-NETA-ePSMA-16 showed that the tumor uptake of the compound was PSMA-specific (1.62 ± 0.55% ID/g, 0.89 ± 0.58% ID/g at 1 and 4 h p.i., respectively). Blood uptake was low at the earliest time-point (0.26 ± 0.19% ID/g, 1 h p.i.), suggesting that the compound is quickly cleared from circulation. Uptake was observed in PSMA expressing organs at 1 h p.i., such as the spleen and prostate, but was rapidly washed out. In the intestines, also known to express PSMA [24], a low but persistent uptake was also seen up to 4 h p.i. (0.60 ± 0.28% ID/g). Barely any uptake was observed in the liver, but a high kidney uptake was observed up to 24 h, characteristic of PSMA-targeting compounds. While kidney uptake was slightly reduced by blocking (11.62 ± 0.62% ID/g vs. 9.53 ± 1.66% ID/g), it remained elevated, suggesting that the uptake was not PSMA-mediated. Nevertheless, it was expected as the kidneys are the main route of excretion for small molecules. The SPECT/CT further corroborated these results at 1 h p.i., where an elevated bladder and kidney uptake was observed. Due to the elevated kidney-to-tumor ratio, barely any signal was observed in the tumor. Dynamic PET/CT imaging with [^18^F]AlF-3p-C-NETA-ePSMA-16 provided a similar biodistribution profile as observed with the SPECT/CT scans of [^111^In]In-3p-*C*-NETA-ePSMA-16. The probe accumulated in the PSMA-positive tumor, and elevated kidney and bladder uptake were observed at early time points. Persistent retention of the probe in the kidneys was observed in both models, leading to elevated kidney-to-tumor ratios. While the tumor uptake for the indium-labeled probe peaked at 1 h and was completely cleared at 24 h p.i., the uptake was retained during the whole duration of the shorter PET/CT experiments for the fluorinated radiotracer. Furthermore, no uptake was observed in the PSMA-negative tumors, confirming that the tumor uptake was specific. The PET/CT scans showed a better contrast in the tumor compared to the SPECT/CT scans; however, the tumor uptake was still relatively low and heterogeneous.

The fast clearance of [^111^In]In-3p-*C*-NETA-ePSMA-16 from the tumor suggests that due to the short biological half-life of this compound, compared to the long half-life of the employed radioisotopes, ^111^In (t_1/2_ = 2.8 d) and ^177^Lu (t_1/2_ = 6.65 d) may not be suitable radiometals for clinical applications. Instead, the shorter-lived ^18^F (t_1/2_ = 109.7 min) and ^213^Bi (t_1/2_ = 45.6 min) would be more suitable candidates. The high and persistent kidney uptake is not desirable due to the radiotoxicity but also because it may hinder tumor identification due to the high background. Furthermore, prolonged tumor retention would be a favorable characteristic for therapeutic applications. Alternatively, strategies to increase the biological half-life of the probe for therapeutic applications may be employed, such as the use of albumin-binding groups, which has shown promise preclinically thus far [25].

Kelly et al. [17] reported the 3p-*C*-DEPA containing [^68^Ga]Ga-EuK-107, which showed good tumor targeting in vivo in a proof-of-concept microPET/CT study in LNCaP tumor xenograft mice, with similar uptake compared to the commercially used [^68^Ga]Ga-DKFZ-PSMA-11 ([^68^Ga]Ga-PSMA-11, Gallium (^68^Ga) gozetotide). This data further supports the potential of this class of hybrid chelators as clinical agents. As we have shown that 3p-*C*-NETA can efficiently complex elements with a smaller ionic radius, such as Al^18^F, this gives rise to the question of whether Al^18^F labeled imaging agents complexed to 3p-*C*-NETA could also be comparable to currently available ^18^F-labeled PSMA-targeting agents, such as the commercially available Pylarify^®^ ([^18^F]DCFPyL, piflufolastat F 18).

Finally, one limitation of our study is the use of theranostic pairs consisting of different elements. While the effect might be minimal, the choice of metal can influence the binding affinity and the biodistribution of the radiotracer [8]. In our previous study [14], we evaluated and compared the binding affinity of [^nat^Tb]Tb-3p-*C*-NETA-TATE, [^nat^Bi]Bi-3p-*C*-NETA-TATE, [^nat^Lu]Lu-3p-*C*-NETA-TATE and AlF-3p-*C*-NETA-TATE. All compounds retained good binding to SSTR2, but affinity values ranged from 15.4 nM ([^nat^Lu]Lu-3p-*C*-NETA-TATE) to 56.0 nM ([^nat^Bi]Bi-3p-*C*-NETA-TATE). This is also why we evaluated the radiochemical properties of ^111^In-, ^177^Lu-, ^213^Bi-, and Al^18^F- labeled 3p-*C*-NETA-ePSMA-16 separately. An alternative to this laborious process would be the use of different isotopes of the same nuclide for imaging or therapy. Such an approach has shown promise so far with the theranostic pair ^64/67^Cu, where the positron-emitting ^64^Cu can be used for PET imaging and the β-emitter ^67^Cu, for therapy [26]. Recently, 3p-*C*-NETA was shown to efficiently complex ^161^Tb. Terbium is an emerging element due to the presence of four potential radioisotopes for nuclear medicine applications [15]. Attached to an optimized targeting vector, this could give rise to a promising 3p-*C*-NETA-based true theranostic.

## 4. Materials and Methods

### 4.1. General Methods

All chemicals and solvents were obtained in reagent grade or better from commercial suppliers and were used without further purification. Fmoc-based solid phase peptide synthesis (SPPS) strategy was employed to synthesize the PSMA moiety and the linker. PSMA-617 was synthesized according to the literature [1]. Chemical reactions were monitored by liquid chromatography-mass spectrometry (LC-MS). An Agilent 1260 Infinity II LC/MSD XT system (Amstelveen, The Netherlands) was employed, together with an Agilent Infinity Lab Poroshell 120 EC-C_18_ column (3 × 100 mm, 2.7 μm). The mobile phase consisted of solvent A, 0.1% formic acid (FA) in water (H_2_O), and solvent B, 0.1% FA in acetonitrile (ACN). The LC gradient used for all analyses was the following: 0–5 min, 5–100% B; 5–8 min, 100% B; at a flow rate of 0.5 mL/min. Electrospray ionization (ESI) in positive mode was used to confirm the identity of the products. Purifications were performed by reverse-phase high-performance liquid chromatography (RP-HPLC) on an Agilent 1290 Infinity II system and an Agilent 5 Prep C_18_ column (50 × 21.2 mm, 5 μm), with solvents A and B as the mobile phase. The following gradient was used: 0–8 min, 5–100% B; 8–11 min, 100% B; at a flow rate of 10 mL/min. The eluents were monitored at 220 or 254 nm, and data were interpreted using the Agilent OpenLab CDS Chemstation software. [^111^In]InCl_3_ was purchased from Curium Netherlands BV (Petten, The Netherlands). [^177^Lu]LuCl_3_ (LuMark^®^) was purchased from IDB Holland (Baarle-Nassau, the Netherlands). ^18^F was produced by bombardment of H_2_^18^O with 18-MeV protons using a cyclotron (IBA Cyclone 18/9, IBA, Louvain-la-Neuve, Belgium). [^213^Bi]BiI_5_^2−^ (0.1 M HCl/0.1 M NaI) was produced onsite from a ^225^Ac/^213^Bi generator provided by the European Commission, Joint Research Centre, Institute for Transuranium Elements (Karlsruhe, Germany).

#### 4.1.1. Quality Control of ^111^In and ^177^Lu Labelings

Instant thin-layer chromatography plates (Agilent iTLC-SG, Folsom, CA, USA) were analyzed using a bSCAN radio-chromatography scanner (Brightspec, Antwerp, Belgium). Radio-HPLC was performed with a Waters Alliance e2695 system (Etten-Leur, The Netherlands) equipped with a 2998 diode array (PDA) detector and a 1-inch NaI crystal radio-detector from Canberra (Zadik, Belgium), using Empower3 software. The analytical Gemini C_18_ column (250 × 4.6 mm, 5 μm) from Phenomenex (Torrance, CA, USA) was used. The mobile phase consisted of solvent C, 0.1% trifluoroacetic acid (TFA) in H_2_O, and solvent D, 0.1% TFA in ACN, at a flow rate of 1 mL/min. The following gradient of solvents C and D was applied: 0–3 min, 5% D; 3–23 min, 5–100% D; 23–27 min, 100% D.

#### 4.1.2. Quality Control of ^213^Bi and Al^18^F Labelings

The radiochemical conversion (RCC) of [^213^Bi]Bi-3p-C-NETA-ePSMA-16 was evaluated by iTLC-SG (Varian, Diegem, Belgium). iTLC-SG papers were developed in an elution chamber using ACN:H_2_O (75/25 *v*/*v*). Regions of interest on the iTLC strip were counted with a gamma counter (PerkinElmer Wizard 1480, Waltham, MA, USA). RCC of [^213^Bi]Bi-3p-C-NETA-ePSMA-16 and radiochemical purity (RCP) of [^18^F]AlF-3p-C-NETA-ePSMA-16 and [^213^Bi]Bi-3p-C-NETA-ePSMA-16 were validated with radio-HPLC (Shimadzu LC20A HPLC System, Kyoto, Japan) coupled in series to a DAD-UV detector at a wavelength of 220 nm and a shielded 3-inch NaI (Tl) scintillation detector connected to a single channel analyzer (Gabi box, Elysia-Raytest, Straubenhardt, Germany). A C_18_ Waters XBridge^®^ column (3.0 × 100 mm, 3.5 µm) was used. The mobile phase consisted of solvent E, 0.05 M ammonium acetate (pH 5.5), and solvent F, ACN, at a flow rate of 0.8 mL/min. The following gradient of solvents E and F was applied: 0–5 min, 5% F; 5–25 min, 20–25% F. Recovery of [^18^F]AlF and [^18^F]F^−^ on this system was previously reported by Tshibangu et al. and was >95% [27,28].

### 4.2. Chemistry

#### 4.2.1. Synthesis of EuK(Ahx-Sta-Phe-Asp) (**1**)

The PSMA-targeting moiety glutamate-urea-lysine (EuK) was synthesized as reported in the literature [29]. Elongation in solid phase was performed by successive coupling of Fmoc-6-Ahx-OH, Fmoc-(3S,4S)Sta-OH, Fmoc-Phe-OH and Fmoc-Asp(tBu)-OH. A scale of 0.1 mmol of 2-chlorotrityl chloride resin (resin loading 0.7 mmol/g, 140 mg resin, 1 equiv.) was used. Amino acids were coupled using 4 equiv. in dimethylformamide (DMF) solution (4 mL) together with 4 equiv. of ethyl cyano(hydroxyimino)acetate (Oxyma pure; 0.4 mmol, 57 mg), 3.92 equiv. of 2-(1*H*-benzotriazol-1-yl)-1,1,3,3-tetramethyluronium hexafluorophosphate (HBTU; 0.392 mmol, 148 mg) and 10 equiv. of *N*,*N*-Diisopropylethylamine (DIPEA; 1.0 mmol, 174 μL). Couplings were performed for 45 min and verified by Kaiser test. Fmoc-deprotection was performed using 20% 4-methyl piperidine in DMF for 15 min and monitored by Kaiser test. After the final Fmoc deprotection, the peptide was cleaved from beads using 4 mL of a TFA/H_2_O/TIPS (95:2.5:2.5; TIPS = triisopropylsilane) cleavage cocktail for 2 h and precipitated using cold diethyl ether. After centrifugation, the crude peptide was recovered and purified by HPLC (*t_R_* = 3.30 min) to afford 21.8 mg (25%) of **1** as a white solid. LC: *t_R_* = 3.62 min, purity > 99%. ESI-MS *m*/*z*: calc’d for C_39_H_61_N_7_O_14_ 851.43; found 852.40 [M + H]^+^.

#### 4.2.2. Synthesis of (4-(4-(5-(4,7-bis(2-(tert-butoxy)-2-oxoethyl)-1,4,7-triazonan-1-yl)-4-(bis(2-(tert-butoxy)-2-oxoethyl)amino)pentyl)phenyl)amino)-4-oxobutanoic acid (3p-*C*-NETA oxanobutanoic acid) (**2**)

The functionalized chelator was synthesized as previously described [14]. LC: *t_R_* = 5.87 min, purity = 97%. ESI-MS m/z: calc’d for C_45_H_75_N_6_O_11_ 861.55; found 862.50 [M + H]^+^.

#### 4.2.3. Synthesis of EuK(Ahx-Sta-Phe-Asp-3p-*C*-NETA(tBu)_4_) (**3**)

To **1** (13.3 mg, 15.6 µmol, 1.5 equiv.) was added **2** (9.0 mg, 10.4 µmol), 1-[bis(dimethylamino)methylene]-1H-1,2,3-triazolo [4,5-b]pyridinium 3-oxide hexafluorophosphate (HATU) (8.0 mg, 10.4 µmol, 1 equiv.) and DIPEA (4 µL, 41.6 µmol, 4 equiv.) in 1 mL of DMF. The reaction was stirred at room temperature (rt) and monitored by LC-MS. The reaction was stopped once the presence of **1** was not observed by LC-MS. Then, the solvent was removed under reduced pressure, and the crude residue resolubilized in H_2_O/ACN (1:1). HPLC purification (*t_R_* = 5.86 min) afforded 2.6 mg of **3** (15%) as a white solid. LC: *t_R_* = 5.72 min, purity > 95%. ESI-MS m/z: calc’d for C_84_H_134_N_12_O_24_ 1694.96; found 848.70 [M + 2H]^2+^.

#### 4.2.4. Synthesis of EuK(Ahx-Sta-Phe-Asp-3p-*C*-NETA), (3p-*C*-NETA-ePSMA-16)

**Scheme 1:**
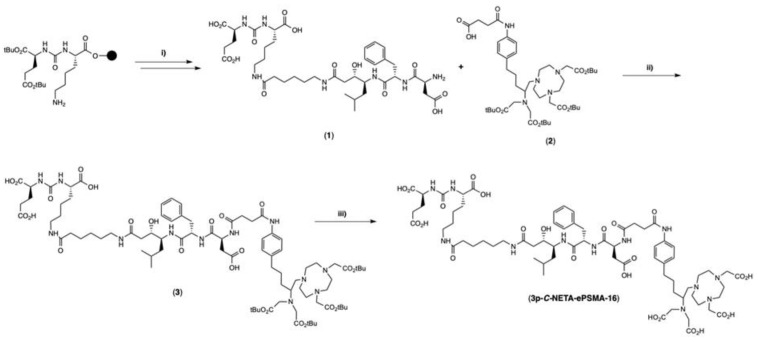
Synthesis of 3p-*C*-NETA-ePSMA-16. The resin used for the SPPS is indicated as a sphere in the scheme. Reagents and conditions: (i) Elongation of the peptide chain with Fmoc-6-Ahx-OH, Fmoc-(3S,4S)Sta-OH, Fmoc-Phe-OH and Fmoc-Asp(tBu)-OH. Each coupling is performed using HBTU, Oxyma, DIPEA, and DMF as a solvent. The peptide is cleaved from the resin using a TFA/H_2_O/TIPS (95:2.5:2.5) cleavage cocktail; (ii) HATU, DIPEA, DMF, rt, 1 h; (iii) TFA/H_2_O/TIPS (95:2.5:2.5), rt, 4 h.

*Tert*-butyl deprotection of **3** was performed by treatment with a solution of TFA/H_2_O/TIPS (95:2.5:2.5, 2 mL) until complete removal of the protective groups was determined by LC-MS. The solvent was removed using a gentle airflow, and the crude residue was resolubilized in H_2_O/ACN (1:1). HPLC purification (*t_R_* = 3.66 min) afforded 1.5 mg of 3p-*C*-NETA-ePSMA-16 (65%) as a white solid (Scheme 1). LC: *t_R_* = 3.81 min, purity > 95%. ESI-MS *m*/*z*: calc’d for C_68_H_102_N_12_O_24_ 1470.71; found 1471.80 [M + H]^+^.

### 4.3. Radiochemistry

#### 4.3.1. Radiolabeling with [^111^In]InCl_3_ and [^177^Lu]LuCl_3_

The concentration of 3p-*C*-NETA-ePSMA-16 was determined prior to labeling via titration, according to a method previously described [30]. A solution containing sodium acetate (1 μL, 2.5 M), ascorbic acid, gentisic acids, and L-methionine (10 μL, 50 mM each) together with Milli-Q water (final volume: 140 µL) was used to perform all labelings [31,32]. 3p-*C*-NETA-ePSMA-16 (1 nmol) dissolved in H_2_O was added to the solution, followed by [^111^In]InCl_3_ (50 MBq, 370 MBq/mL) or [^177^Lu]LuCl_3_ (50 MBq, 2 GBq/150 μL). The mixture was incubated for 5 min at rt (^111^In) or at 90 °C (^177^Lu). iTLC-SG strips eluted with a solution of sodium citrate (0.1 M, pH 5.0) were employed for quality control, and free radiometal was complexed by adding DTPA (5 μL, 4 mM) before injection onto radio-HPLC.

#### 4.3.2. Radiolabeling with ^213^BiI_5_^2−^

^213^Bi was milked from the ^225^Ac/^213^Bi generator (22–24 MBq), as reported in the literature [33]. Briefly, ^213^BiI_5_^2−^ was eluted using a mixture of 300 µL of 0.2 M HCl and 300 µL of 0.2 M NaI directly into a vial filled with 100 µL 4 M Tris-HCl (pH 8.5). Elution was performed at 3 h intervals, and the generator was always stored in 0.01 M HCl. The synthesis of [^213^Bi]Bi-3p-*C*-NETA-ePSMA-16 was performed by reacting ^213^BiI_5_^2−^ (6–12 MBq) with 3p-*C*-NETA-ePSMA-16 (3 nmol, 300 µL, 10 µM) at 95 °C for 7 min (final volume: 600 µL) under constant shaking. The RCC and RCP were determined using iTLC-SG plates and radio-HPLC. The radiopharmaceutical migrated on the iTLC-SG strips with the solvent front (R_f_ = 0.9–1), while unbound radionuclides remained at the origin (R_f_ = 0.14–0.22) (eluent: ACN/H_2_O, 3:1).

#### 4.3.3. Radiosynthesis of Al [^18^F]F-3p-*C*-NETA-ePSMA-16

[^18^F]AlF-3p-*C*-NETA-ePSMA-16 was radiolabeled in an automated AllinOne^®^ synthesis module (Trasis, Liège, Belgium) using 3p-*C*-NETA-ePSMA-16, in identical conditions as for [^18^F]AlF-3p-*C*-NETA-TATE, as previously described [27].

#### 4.3.4. Radiochemical Stability of [^111^In]In-3p-*C*-NETA-ePSMA-16 and [^177^Lu]Lu-3p-*C*-NETA-ePSMA-16

Stability was determined by mixing 20 μL of the radiolabeled product (~10 MBq) with 80 μL of PBS (0.01 M, pH 7.4) or 70 μL of radiolabeled 3p-*C*-NETA-ePSMA-16 (~30 MBq) with 330 µL of mouse serum. The mixture was incubated at 37 °C for up to 24 h in an Eppendorf ThermoMixer C (Enfield, CT, USA). Then, 50 μL of the mouse serum solution was added to a separate Eppendorf vial containing an equal volume of ACN after incubation. The sample was vortexed and centrifuged at 5000× *g* for 20 min. The supernatant was separated and analyzed by radio-HPLC. The PBS solution was directly injected onto the radio-HPLC. Stability was monitored at 1, 4, and 24 h.

#### 4.3.5. Radiochemical Stability of [^213^Bi]Bi-3p-*C*-NETA-ePSMA-16 and [^18^F]AlF-3p-*C*-NETA-ePSMA-16

A sample of 50 µL of [^213^Bi]Bi-3p-*C*-NETA-ePSMA-16 or [^18^F]AlF-3p-*C*-NETA-ePSMA-16 (5–10 MBq) was added to a 1 mL vial containing 450 µL of human serum in triplicate. The solution was incubated at 37 °C under constant gentle shaking. The percentage (%) of intact [^213^Bi]Bi-3p-*C*-NETA-ePSMA-16 or [^18^F]AlF-3p-*C*-NETA-ePSMA-16 was determined by iTLC analysis and confirmed with radio-HPLC at the last time point as described above. The stability was monitored at 10, 30, 60, 120, and 240 min.

#### 4.3.6. Determination of LogD_7.4_ Value

The distribution coefficient (LogD_7.4_) was determined by a shake-flask method in triplicate. [^111^In]In-3p-*C*-NETA-ePSMA-16 (~0.5 MBq) was added to a solution of PBS (500 μL, 0.01 M, pH 7.4) and n-octanol (500 μL). The mixture was vortexed vigorously and then centrifuged at 5000× *g* for 15 min for phase separation. Samples of the two phases (3 × 10 μL) were taken out and measured using a PerkinElmer Wizard 2 γ-counter. LogD_7.4_ values were calculated using the following equation: LogD_7.4_ = log [(counts in octanol phase)/(counts in aqueous phase)] [34].

#### 4.3.7. Transchelation/Challenge Studies

Transchelation studies were performed by incubating 20 µL of the labeling solution (~10 MBq) of [^111^In]In-3p-*C*-NETA-ePSMA-16 with 20 µL of EDTA solution (34 mM in 0.1 M PBS) at 37 °C in triplicate. Samples were analyzed by iTLC at 1, 4, and 24 h after incubation to determine the percentage (%) of intact radioligand [16].

### 4.4. Biological Assays

#### 4.4.1. NAALADase Assay

The binding affinity of 3p-*C*-NETA-ePSMA-16 was determined enzymatically using a fluorescence-based NAALADase enzymatic assay. The assay buffer (50 mM HEPES, 0.1 M NaCl, pH 7.5) was used to dilute the recombinant human PSMA (rhPSMA, R&D systems, Abingdon, UK) to 0.4 µg/mL, as well as the enzymatic substrate Ac-Asp-Glu (NAAG) (40 μM in assay buffer). 3p-*C*-NETA-ePSMA-16 and an internal reference, PSMA-617, were dissolved in assay buffer at concentrations ranging from 10^−5^ to 10^−12^ M. 6.25 µL of the compound were incubated with 6.25 µL of the substrate to which 12.5 µL of rhPSMA solution was added, and incubated for 1 h at 37 °C in 384-well black polystyrene microplates. Next, 25 µL of a working solution of the Amplex Red glutamic acid kit (ThermoFisher, Bleiswijk, The Netherlands) was added and incubated at 37 °C for 30 min to measure glutamate released. A HIDEX Sense Optical System (HIDEX, Goedereede, The Netherlands) was used to measure the fluorescence, with excitation set at 535 nm and emission at 590 nm. Assays were performed in triplicate and as three separate replicates. Data were normalized and analyzed using a one-site total binding regression algorithm from GraphPad Prism 9 (GraphPad Software, Boston, MA, USA).

#### 4.4.2. Cell Lines

LS174T human colon carcinoma cells transfected with human PSMA were provided by Dr. Sandra Heskamp (Radboudumc, Nijmegen, The Netherlands). RPMI-1640 media supplemented with 2 mM glutamine, 10% fetal bovine serum (FBS), and 0.3 mg/mL G418 (Geneticin, Sigma Aldrich, Burlington, MA, USA) was used to culture the cells. PC-3-Pip and PC-3-Flu cells were received from Prof. Martin G. Pomper (John Hopkins University, Baltimore, MD, USA).

#### 4.4.3. Cell Uptake and Internalization Assay

LS174T cells were seeded (1.2 × 10^6^ cells/well) and cultured to confluency 24 h prior to the experiments. [^111^In]In-PSMA-617 and [^111^In]In-3p-*C*-NETA-ePSMA-16 were prepared as 10^−9^ M solutions in internalization media (RPMI, 20 mM HEPES, 1% BSA, pH 7.4). [^111^In]In-PSMA-617 was used as an internal standard. Blocking experiments were also performed using a 50-fold excess of either PSMA-617 or 3p-*C*-NETA-ePSMA-16. The media was removed, and wells were then rinsed twice with PBS at rt. To each well, we added 2 mL of the radioactive compound, which was incubated at 37 °C for 2 h. Internalization assay: the media containing the radioactive compound was removed from each well, followed by two washes with 1 mL cold PBS. Glycine buffer (1 mL, 50 mM glycine, 100 mM NaCl, pH 2.8) was immediately added to the wells prior to incubation at rt for 10 min. The glycine wash and an additional wash were collected into counting tubes (membrane-bound fraction). NaOH (1 mL, 1 M) was added to each well for 15 min at rt. The internalized fraction (lysate) and an additional NaOH wash were collected in the same tubes. Data were normalized to the added dose per 1,000,000 cells (AD/10^6^ cells) and represented as counts per minute (CPM).

#### 4.4.4. Animal Model for SPECT/CT

We housed 8–10 weeks old male BALB/c nude mice (BALB/cAnN Rj-Nude; Janvier Labs, Le Genest-Saint-Isle, France) in individually ventilated cages (Blue line IVC, 2 mice per cage). Sterile standard conditions were employed with the presence of cage enrichment and unlimited access to water and animal chow (Sniff GmbH). Acclimatization was realized 1 week before the beginning of the experiments. Mice were subcutaneously inoculated in their right flank with LS174T cells (4.0 × 10^6^) diluted in 100 µL of cell suspension. Tumors grew to an average volume of 217.8 ± 9.4 mm^3^ in 7 days (average weight of 217 ± 14.4 mg), and mice were grouped non-blinded to afford homogenous tumor size distribution intra-groups. [^111^In]In-3p-*C*-NETA-ePSMA-16 was injected intravenously via the tail vein 7 days after tumor cell inoculation. All experiments were in accordance with the guidelines of the Revised Dutch Act on Animal Experimentation (WOD).

#### 4.4.5. Animal Model for PET/CT

We housed 6–8-week-old female SCID mice (SCID Beige C B17.Cg-Prkdc scid Lystbg-J/Crl; Charles River Laboratories, Sulzfeld, Germany) in individually ventilated cages in a humidity-controlled facility at approx. 22 °C and with a 12 h-12 h light-dark cycle and unlimited access to food and water. The subcutaneous tumor xenograft model was prepared following a published procedure [35]. Briefly, PC3-Pip cells (1 × 10^6^) mixed with Cultrex (1:1; Cultrex Basement Membrane Extract, R&D systems, Minneapolis, USA) were implanted subcutaneously into the right shoulder of the mice. PC3-flu cells were prepared under the same conditions and injected into the left shoulder of the same animals. After an average of four weeks, the mice were used in PET/computed tomography (CT) imaging studies. Tumor volumes ranged from 400 to 600 mm^3^, while the tumor mass-to-body weight ratio was 0.16–2.44%. The animals included in this study were selected randomly among the in-house bred mice of the correct age. No exclusion criteria were used, and all subsequent studies and analyses were conducted unblinded. All animal procedures were approved by the KU Leuven ethical review board (ethical approval reference P200/2021) and were conducted according to Directive 2010/63/EU and reported according to the ARRIVE guidelines [36].

#### 4.4.6. Ex Vivo Biodistribution of [^111^In]In-3p-*C*-NETA-ePSMA-16

Mice were intravenously injected with 5 MBq/1 nmol of [^111^In]In-3p-*C*-NETA-ePSMA-16 for ex vivo biodistribution studies. Mice (*n* = 4/group) were euthanized via cervical dislocation at 1, 4, and 24 h post-injection (p.i.). A block group (*n* = 4) containing a 50-fold excess of 3p-*C*-NETA-ePSMA-16 was also included in the study (euthanized at 4 h p.i.). Tissues were collected in tubes, weighted, and counted in the gamma counter. Various known activities of ^111^In were measured to determine the calibration factor.

#### 4.4.7. SPECT/CT Imaging

Mice were intravenously injected with 20 MBq/1 nmol of [^111^In]In-3p-*C*-NETA-ePSMA-16 (*n* = 4). At 1 and 24 h p.i., mice were placed under 2% isoflurane/O_2_ anesthesia on a heated bed and imaged in a dedicated small-animal PET/SPECT/CT scanner (VECTor5CT scanner, MILabs B.V., Utrecht, The Netherlands) equipped with a high sensitivity pinhole collimator (XXUHS-M, 3.00 mm pinhole diameter). Whole-body SPECT images were acquired over 30 min in list-mode acquisition (using a spiral scan in normal scan mode, a transaxial field of view of 54 mm) followed by a whole-body CT scan (full angle scan, angle step 0.75 degrees, normal scan mode, 50 kV tube voltage, 0.21 mA tube current, 500 µm aluminum filter). Reconstruction of the SPECT images was performed by applying the similarity-regulated SROSEM method and MLMN method (MILabs Rec 12.00 software) with a total of 9 and 128 iterations, respectively, at a resolution of 0.8 mm^3^. Energy windows for ^111^In used were 173 keV ± 10% and 247 keV ± 10%. Two adjacent background windows per photo peak were used for crosstalk correction and triple-energy window scatter. An isotropic 3-dimensional Gaussian filter of 1 mm full width, at half-maximum, was applied to post-filter the reconstructed volumes of the SPECT scans. The CT and registered attenuation-corrected SPECT images were analyzed using IMALYTICS Preclinical 3.0 (Gremse-IT GmbH, Aachen, Germany).

#### 4.4.8. PET/CT Imaging

Small animal whole-body PET imaging was performed by intravenously administering [^18^F]AlF-3p-*C*-NETA-ePSMA-16 (1.5–3.0 MBq) into the tumor-bearing mice. Immediately after injection, dynamic PET images were acquired for 85 min using a β-cube PET scanner (Molecubes, Gent, Belgium). During the entire procedure, mice were kept under gas anesthesia (2.5% isoflurane in O_2_ at 1 L/min flow rate) with monitoring of temperature and respiration. A CT scan (50 kVp, 480 exposures, 85 ms/projection, 100 μA tube current, rotation time 60 s) was acquired for anatomic co-registration with an X-cube CT scanner (Molecubes). PET data were histogrammed into 14 frames (4 × 15 s, 4 × 1 min, 1 × 5 min, 5 × 10 min) and reconstructed using the MLEM algorithm (192 × 192 image matrix, 0.4 mm voxels, 30 iterations) with corrections performed for randoms, scatter, attenuation, and decay. For CT data, a regularized statistical (iterative) image reconstruction algorithm was used for reconstruction, using non-negative least squares and an isotropic 200 μm voxel size scaled to Hounsfield Units (Hus) after calibration against a standard air/water phantom). The fused PET-CT image was displayed using PFUS 4.0 (PMOD Technologies GmbH, Zürich, Switzerland), and volumes of interest were manually drawn over the tumor while a sphere of 3 mm diameter was drawn over the left lobe of the liver.

#### 4.4.9. Statistical Analysis

Statistical analysis was performed using GraphPad Prism 9, and results are represented as mean ± standard deviation (SD). A Grubbs test was used to test for outliers, while Shapiro–Wilk was used to test for normality. An unpaired *t*-test was used to evaluate the competitive binding, uptake and internalization, and biodistribution data for significant differences. Statistically significant differences were considered for *p*-values below 0.05. *p*-values that were smaller than 0.05 (*p* < 0.05), *p* < 0.01, *p* < 0.001 and *p* < 0.0001 were visually indicated by one (*), two (**), three (***) or four (****) asterisks, respectively. 

## 5. Conclusions

3p-*C*-NETA-ePSMA-16 was synthesized in high chemical purity and showed good binding affinity to PSMA, as well as PSMA-specific uptake and internalization. [^111^In]In-3p-*C*-NETA-ePSMA-16 was obtained in mild conditions with high specific activity and remained stable in PBS, mouse serum, and transchelation up to 24 h p.i. Radiolabeling with ^177^Lu, ^213^Bi, and Al^18^F equally afforded high radiochemical yields, purity, and stability, indicating the potential of 3p-*C*-NETA-ePSMA-16 as a radiotracer for imaging (^111^In, ^18^F) and radioligand therapy (^177^Lu, ^213^Bi). Biodistribution studies with [^111^In]In-3p-C-NETA-ePSMA-16 in PSMA-positive LS174T mouse xenograft models showed PSMA-specific tumor uptake and low background uptake, except in the kidneys. The compound was rapidly excreted from the tumor and also presented fast renal clearance. Tumor visualization, however, was faint in SPECT-CT images. PET-CT scans of [^18^F]AlF-3p-*C*-NETA-ePSMA-16 showed uptake of the compound in the tumor during the whole experiment, with low liver uptake and strong signal in the kidneys. The rapid uptake of the probe in the tumor and the better contrast in the PET-CT scans suggest that the compound is more suitable to be used in combination with short-lived radionuclides. Further optimization is nevertheless needed to improve tumor-to-kidney ratios and the pharmacokinetics of this compound. However, our study further illustrates the versatility of 3p-*C*-NETA, and the promising results obtained so far may pave the way for further clinical translation of this chelator.

## Figures and Tables

**Table 1:** Summary of the radiochemical properties of [^111^In]In-3p-*C*-NETA-ePSMA-16, [^177^Lu]Lu-3p-*C*-NETA-ePSMA-16, [^213^Bi]Bi-3p-*C*-NETA-ePSMA-16 and [^18^F]AlF-3p-*C*-NETA-ePSMA-16. RCY was determined by radio-ITLC, while RCP and stability were determined by radio-HPLC.

Radionuclide	RCY	RCP	Stability in PBS	Stability in Serum
^111^In	>99%	>99%	>99%, 24 h	>99%, 24 h ^b^
^177^Lu	93.6%	89%	89%, 24 h	89%, 24 h ^b^
^213^Bi	94.0% ^a^	94.0%	-	97.4%, 4 h ^c,d^
Al^18^F	40% ^e^	97.4%	-	96.1%, 4 h ^c^

^a^ Indicates the radiochemical conversion (RCC) as measured by iTLC. ^b^ Stability was evaluated in mouse serum. ^c^ Stability was evaluated in human serum. ^d^ As a reference for the stability of [^213^Bi]Bi-3p-*C*-NETA-ePSMA-16 in serum, we considered the area of the peak after radiolabeling (RCP of 94%) as 100%. ^e^ Decay-corrected, activity final batch of purified product/activity in reactor.

## Data Availability

The data present in this study are available in the body of the manuscript and in the Supplementary Materials.

## Supplementary Materials

The following supporting information can be downloaded at: https://www.mdpi.com/article/10.3390/ph16060882/s1, Figure S1: Characterization of 1–4; Figure S2: Stability of [^111^In]In-3p-C-NETA-ePSMA-16 in PBS at 37 °C for up to 24 h; Figure S3: Stability of [^111^In]In-3p-C-NETA-ePSMA-16 in mouse serum at 37 °C for up to 24 h; Figure S4: Transchelation of [^111^In]In-3p-*C*-NETA-ePSMA-16 with 1000-fold excess EDTA; Figure S5: Radiochemical yield (RCY) of the labeling of [^111^In]In-3p-*C*-NETA-ePSMA-16 with different precursor amounts (nmol); Figure S6: Stability of [^177^Lu]Lu-3p-*C*-NETA-ePSMA-16 in PBS at 37 °C for up to 24 h; Figure S7: Stability of [^177^Lu]Lu-3p-*C*-NETA-ePSMA-16 in mouse serum at 37 °C for up to 24 h; Figure S8: Stability of [^213^Bi]Bi-3p-*C*-NETA-ePSMA-16 in human serum for up to 4 h; Figure S9: Stability of Al [^18^F]F-3p-*C*-NETA-ePSMA-16 in human serum for up to 4 h; Table S1: Ex vivo biodistribution of [^111^In]In-3p-C-NETA-ePSMA-16; Table S2: In vivo biodistribution of [^18^F]AlF-3p-C-NETA-ePSMA-16.
