# Supplementary material for: Radiochemical and Biological Evaluation of 3p-C-NETA-ePSMA-16, a Promising PSMA-Targeting Agent for Radiotheranostics
Source: Pharmaceuticals (Basel). 2023 Jun 15;16(6):882. doi: 10.3390/ph16060882 (PMC10301001; doi:10.3390/ph16060882)
Supplement: Supplementary file 1 [file pharmaceuticals-16-00882-s001.zip › pharmaceuticals-2429490-supplementary.pdf]

Supplementary Information

Radiochemical and Biological Evaluation of 3p-*C*-NETA-ePSMA-16, a Promising PSMA-Targeting Agent for Radiotheranostics

Erika Murce [1,2], Stephen Ahenkorah [3,4], Savanne Beekman [1,2], Maryana Handula [1,2], Debra Stuurman [1,2], Corrina de Ridder [1,2], Frederik Cleeren [4] and Yann Seimbille [1,2,5,*]

1 Department of Radiology and Nuclear Medicine, University Medical Center, Rotterdam, Erasmus MC, 3015 GD Rotterdam, The Netherlands; e.murcesilva@erasmusmc.nl (E.M.); s.beekman@erasmusmc.nl (S.B.); m.handula@erasmusmc.nl (M.H.); d.stuurman@erasmusmc.nl (D.S.); c.deridder@erasmusmc.nl (C.d.R.)
2 Erasmus MC Cancer Institute, 3015 GD Rotterdam, The Netherlands
3 NURA research group, Belgian Nuclear Research Center (SCK CEN), 2400 Mol, Belgium; stephen.ahenkorah@kuleuven.be
4 Radiopharmaceutical Research, Department of Pharmacy and Pharmacology, University of Leuven, 3000 Leuven, Belgium; frederik.cleeren@kuleuven.be
5 TRIUMF, Life Sciences Division, Vancouver, BC V6T 2A3, Canada
* Correspondence: y.seimbille@erasmusmc.nl; Tel.: +31-10-703-8961

Table of Contents

CHARACTERIZATION OF 1-4 2
STABILITY OF [^{111}IN]IN-3P-C-NETA-EPSMA-16 4
STABILITY OF [^{177}LU]LU-3P-C-NETA-EPSMA-16 8
STABILITY OF [^{18}F]ALF-3P-C-NETA-EPSMA-16 11
EX VIVO BIODISTRIBUTION OF [^{111}IN]IN-3P-C-NETA-EPSMA-16 12
IN VIVO BIODISTRIBUTION OF [^{18}F]ALF-3P-C-NETA-EPSMA-16 13

Characterization of 1-4

Compounds **1-4** were characterized for their chemical purity by LC-MS at 220 or 254 nm and for their identity by ESI-MS.

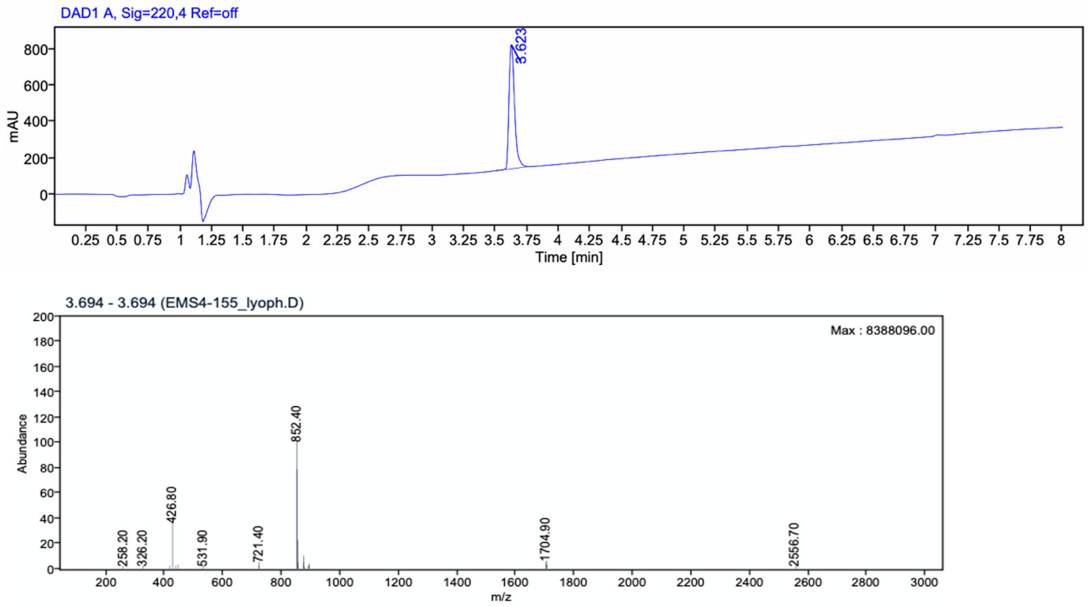

Figure S1.1: LC chromatogram and mass spectrum of **1**. ESI-MS m/z: calc'd for $C_{39}H_{61}N_7O_{14}$ 851.43; found 852.40 $[M+H]^+$.

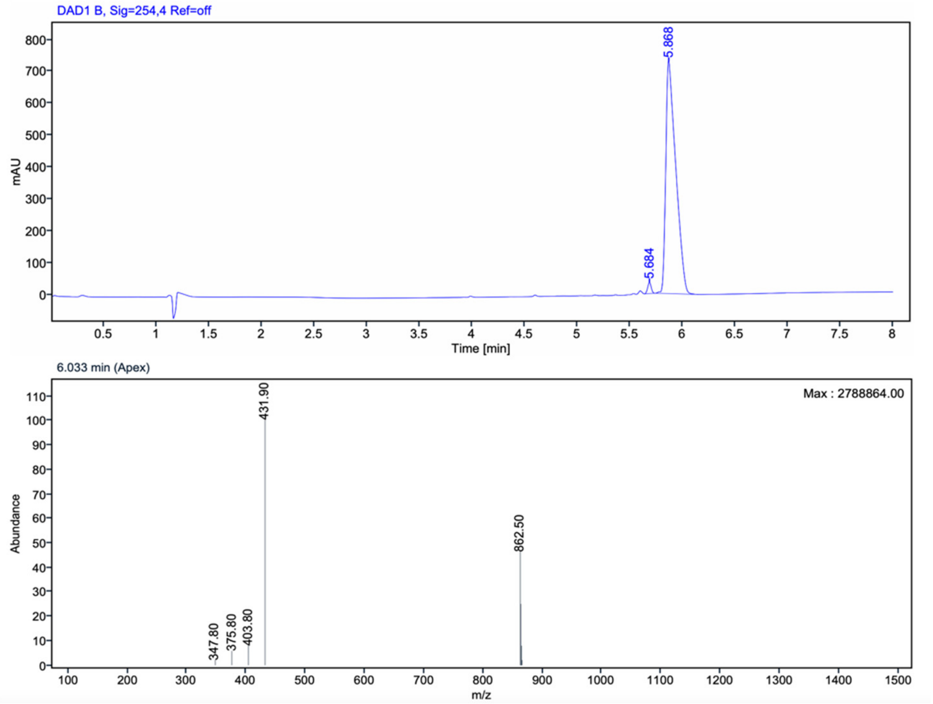

Figure S1.2: LC chromatogram and mass spectrum of **2**. ESI-MS m/z: calc'd for $C_{45}H_{75}N_6O_{11}$ 861.55; found 862.50 $[M+H]^+$.

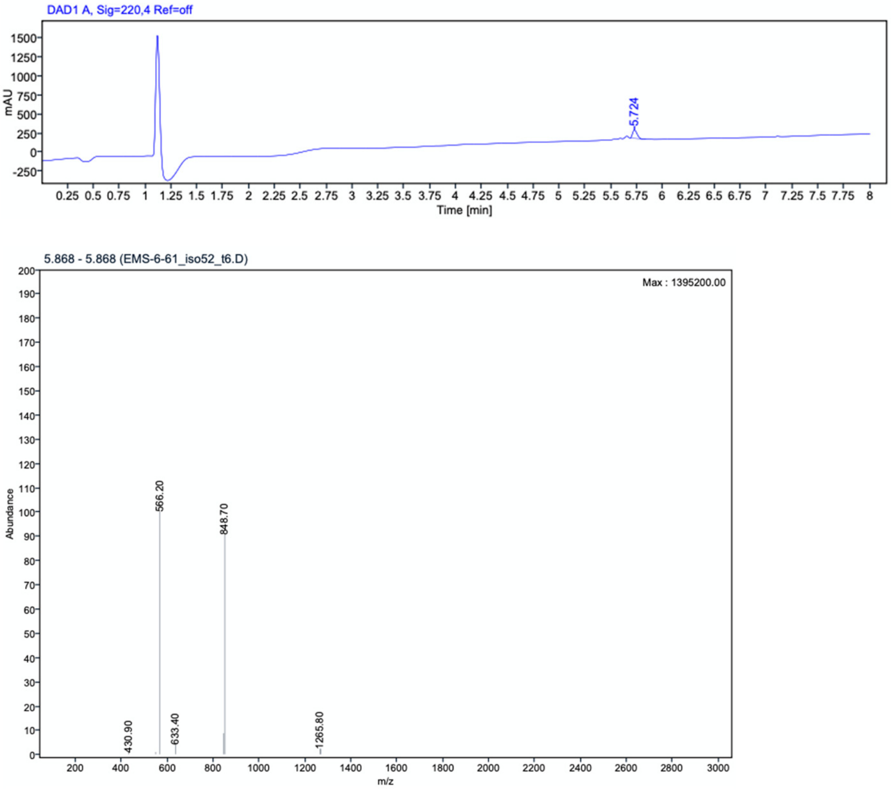

Figure S1.3: LC chromatogram and mass spectrum of **3**. ESI-MS m/z: calc'd for $C_{84}H_{134}N_{12}O_{24}$ 1694.96; found 848.70 $[M+2H]^{2+}$; 566.20 $[M+3H]^{3+}$.

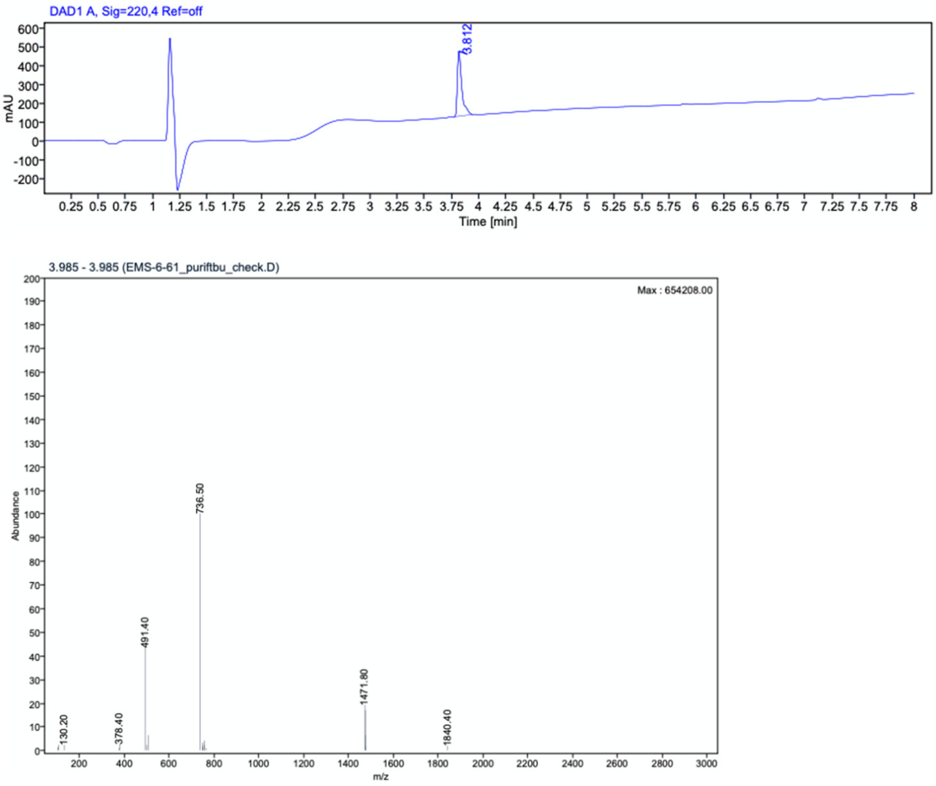

Figure S1.4: LC chromatogram and mass spectrum of **3p-*C*-NETA-ePSMA-16**. ESI-MS m/z: calc'd for $C_{68}H_{102}N_{12}O_{24}$ 1470.71; found 1471.80 $[M+H]^{+}$; 736.50 $[M+2H]^{2+}$.

Stability of [^{111}In]In-3p-C-NETA-ePSMA-16

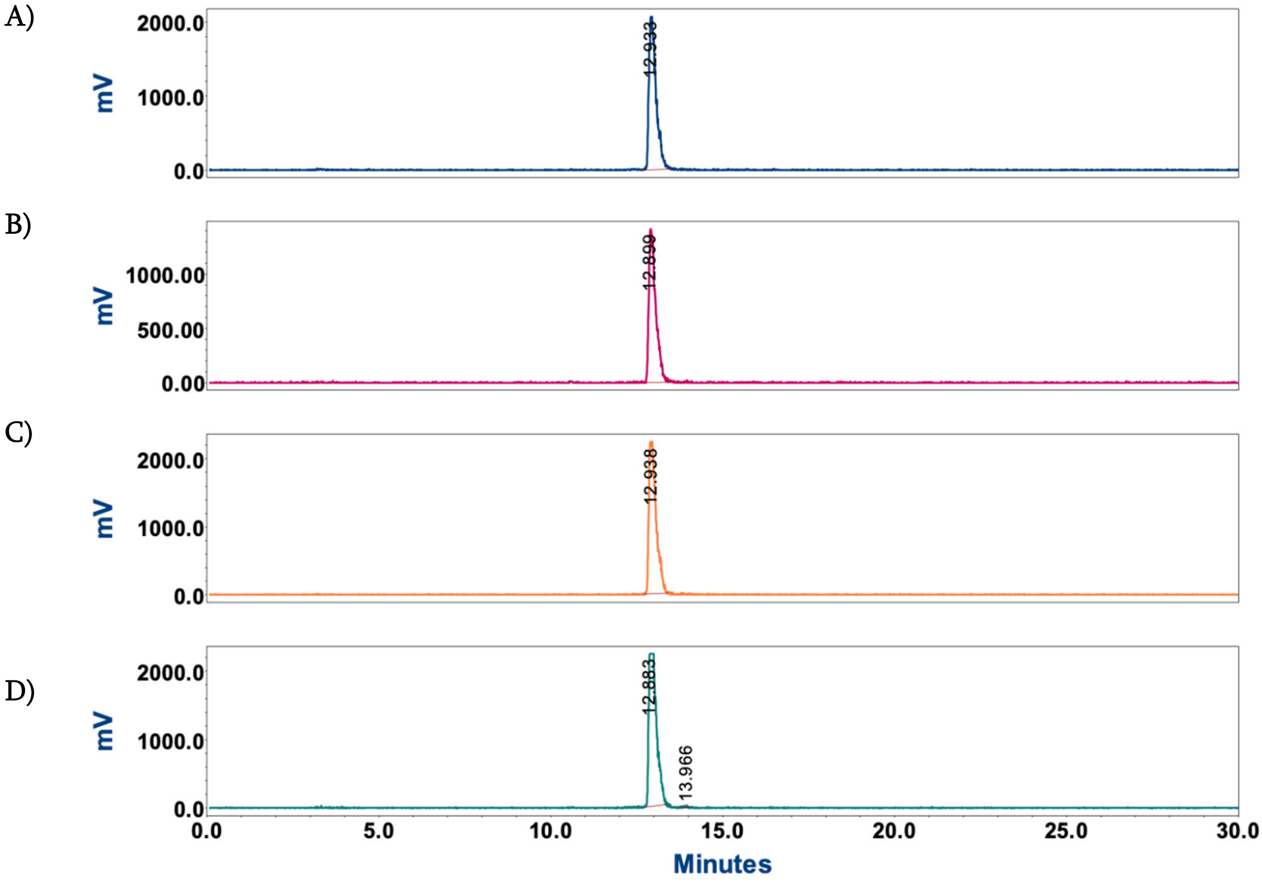

Figure S2. Stability of [^{111}In]In-3p-*C*-NETA-ePSMA-16 in PBS at 37 °C for up to 24 h. (A) radio-HPLC injection of the labeling solution. (B), (C) and (D) radio-HPLC injection of solution after 1, 4 and 24 h incubation in PBS, respectively.

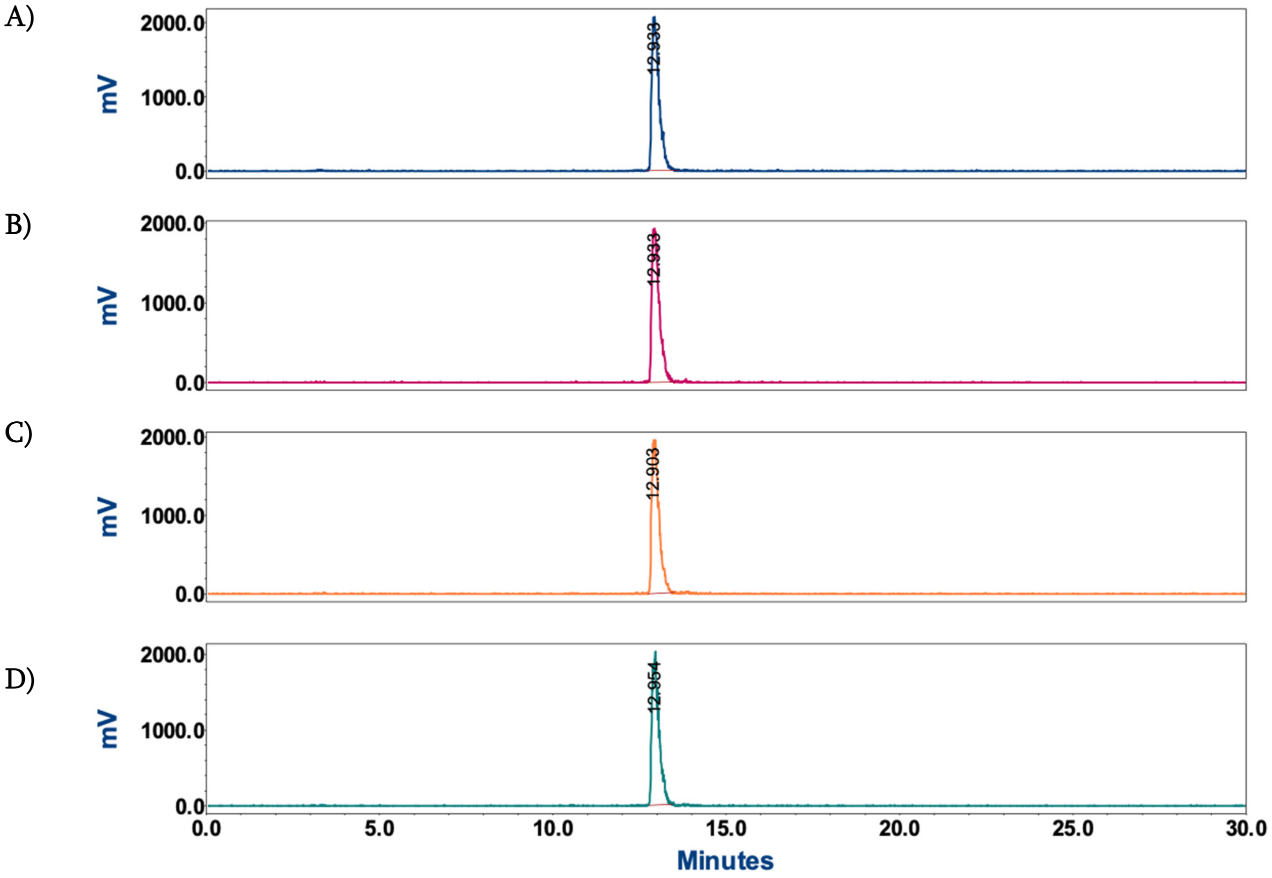

Figure S3. Stability of $[^{111}In]$In-3p-*C*-NETA-ePSMA-16 in mouse serum at 37 °C for up to 24 h. (A) radio-HPLC injection of the labeling solution. (B), (C) and (D) radio-HPLC injection of solution after 1, 4 and 24 h incubation in mouse serum, respectively.

A)

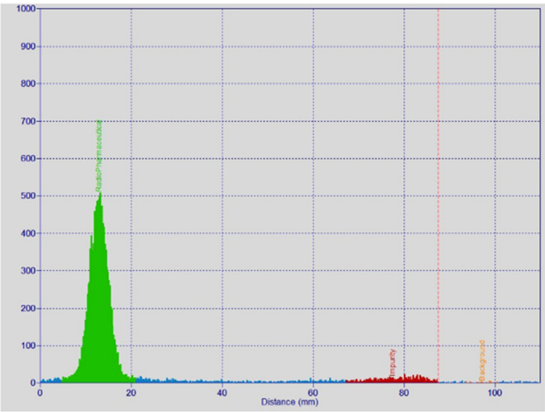

B)

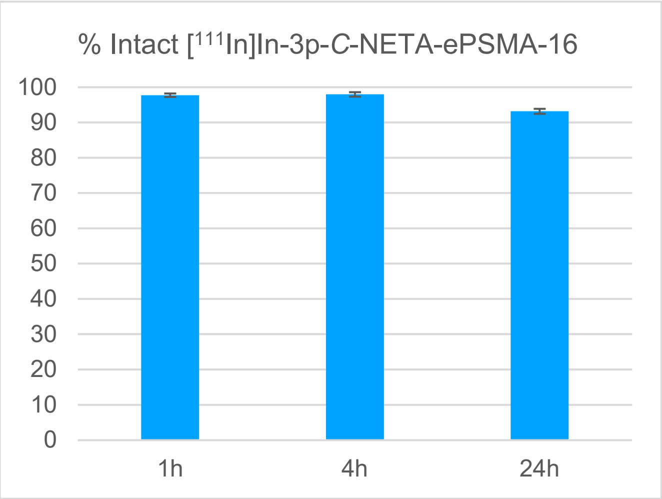

Figure S4. Transchelation of [^{111}In]In-3p-*C*-NETA-ePSMA-16 with 1000-fold excess EDTA. (**A**) iTLC of [^{111}In]In-3p-*C*-NETA-ePSMA-16 after 24 h incubation with excess EDTA at 37 °C. (**B**) Plotted values of the RCY of [^{111}In]In-3p-*C*-NETA-ePSMA-16 after incubation with excess EDTA at 1, 4 and 24 h (n = 3 for each timepoint).

A)

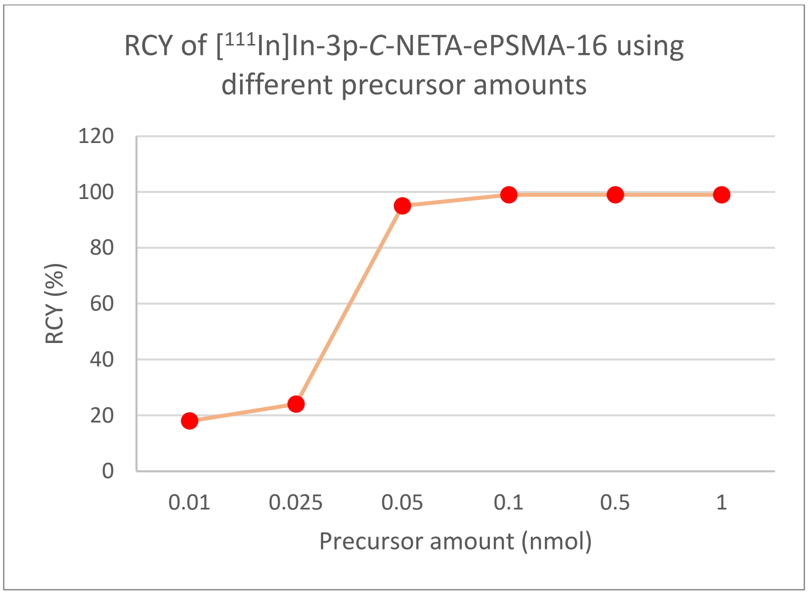

B)

Amount of precursor (nmol)	RCY (%)
1.000	99.9
0.500	99.5
0.100	99.4
0.050	95.4
0.025	24.4
0.010	18.1

Figure S5. Radiochemical yield (RCY) of the labeling of [^{111}In]In-3p-*C*-NETA-ePSMA-16 with different precursor amounts (nmol). A standard SA of 10 MBq was used for all labelings. (**A**) Plotted values of the RCY of [^{111}In]In-3p-*C*-NETA-ePSMA-16 when labeled with different precursor amounts. (**B**) Numerical values of the RCY at the different precursor amounts.

Stability of [^{177}Lu]Lu-3p-C-NETA-ePSMA-16

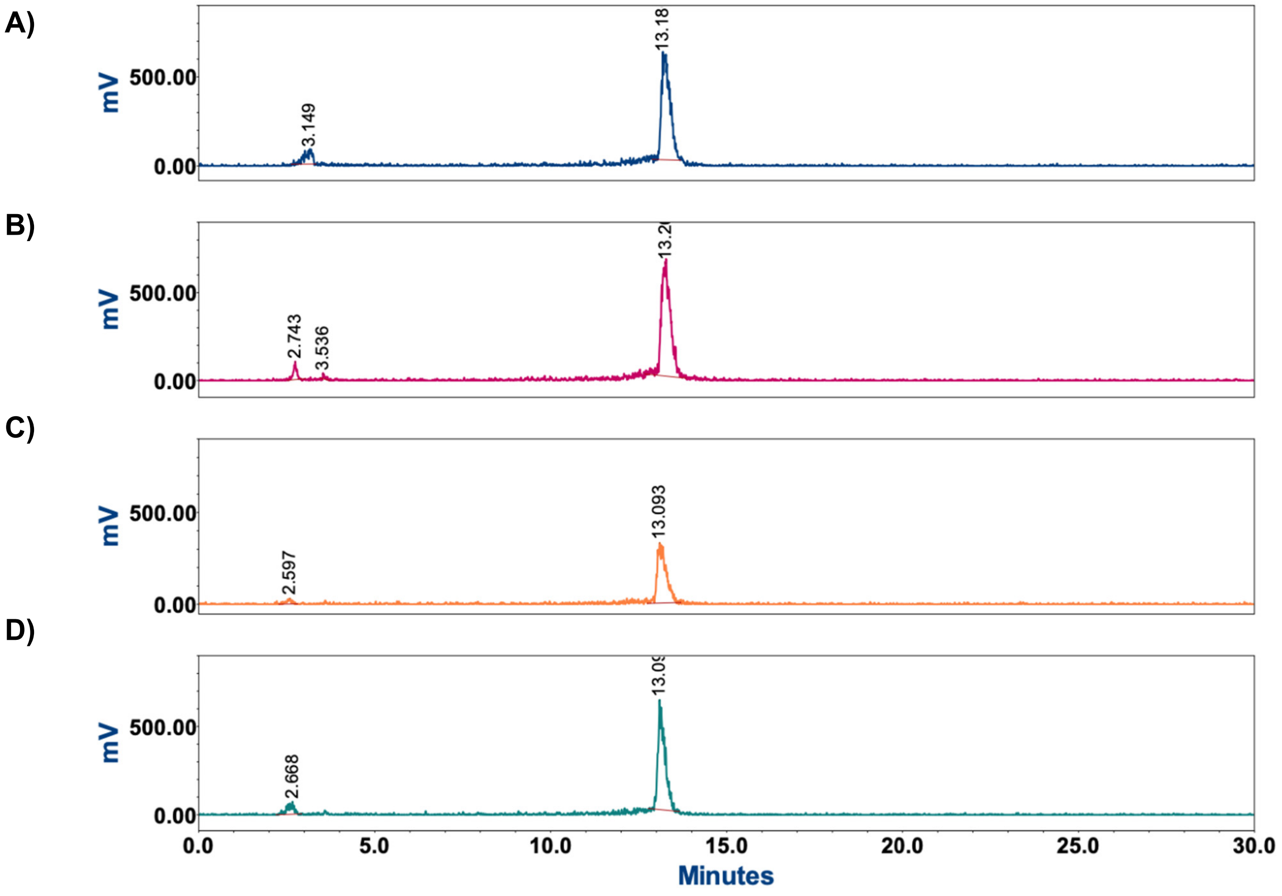

Figure S6. Stability of [^{177}Lu]Lu-3p-*C*-NETA-ePSMA-16 in PBS at 37 °C for up to 24 h. (A) radio-HPLC injection of the labeling solution. (B), (C) and (D) radio-HPLC injection of solution after 1, 4 and 24 h incubation in PBS, respectively.

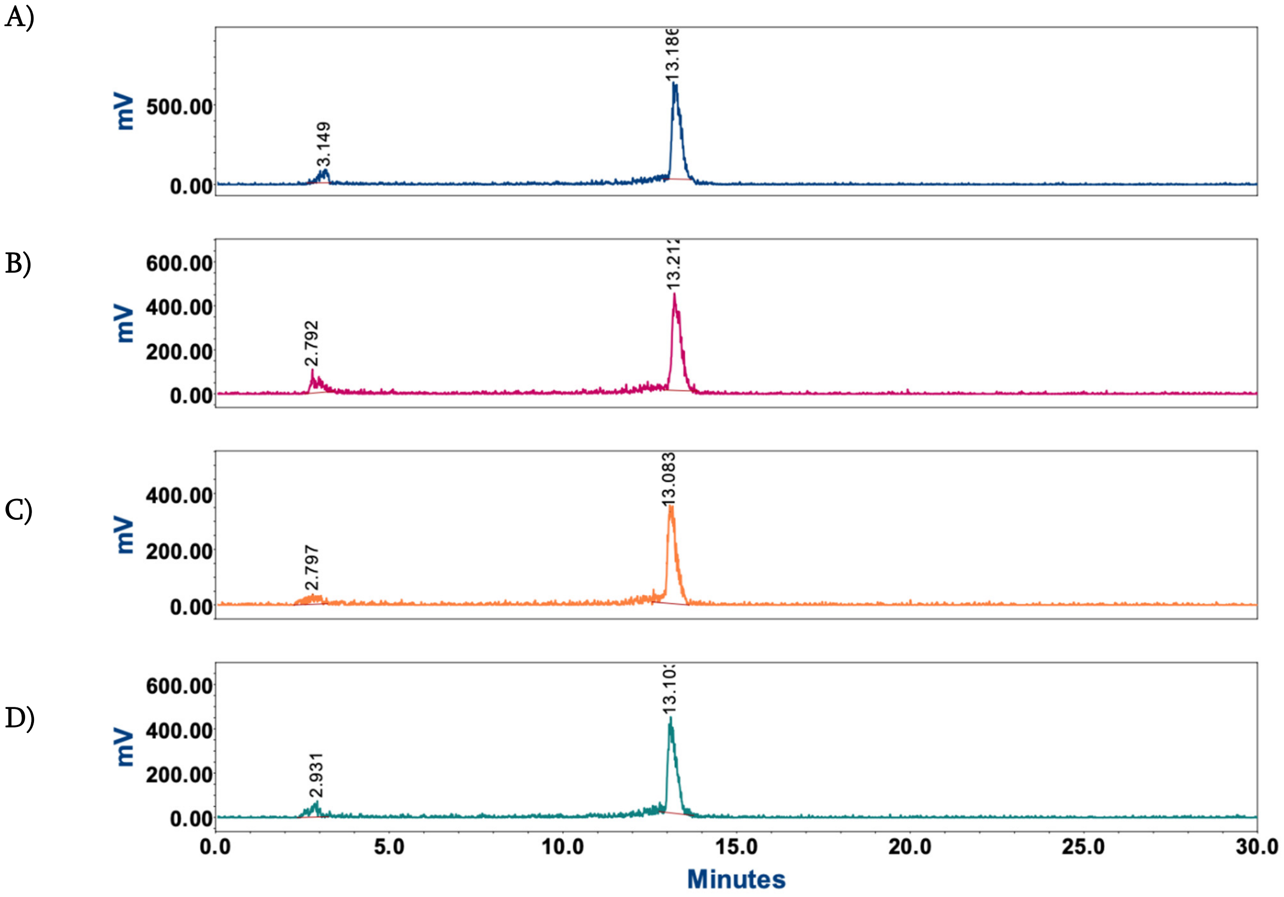

Figure S7. Stability of [^{177}Lu]Lu-3p-*C*-NETA-ePSMA-16 in mouse serum at 37 °C for up to 24 h. (A) radio-HPLC injection of the labeling solution. (B), (C) and (D) radio-HPLC injection of solution after 1, 4 and 24 h incubation in mouse serum, respectively.

Stability of [^{213}Bi]Bi-3p-C-NETA-ePSMA-16

A)

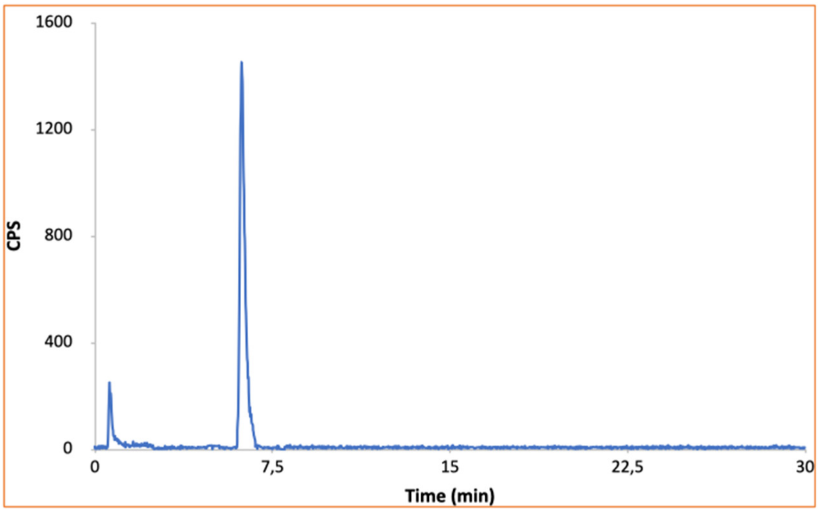

B)

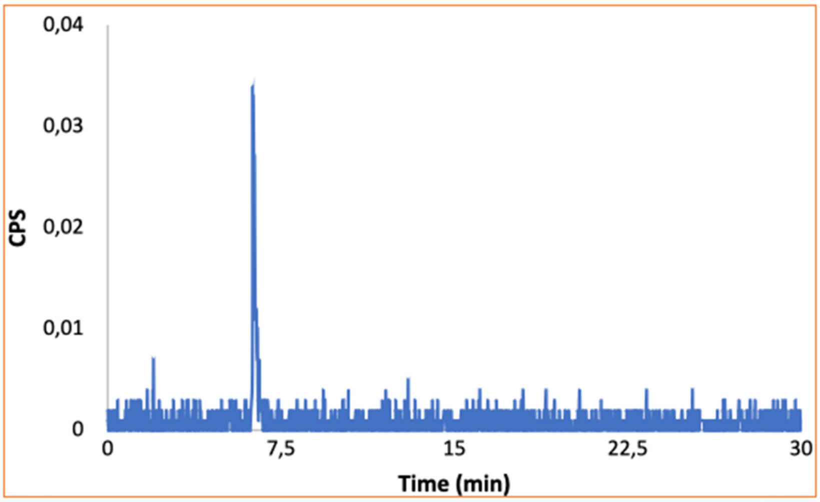

Figure S8. Stability of [^{213}Bi]Bi-3p-*C*-NETA-ePSMA-16 in human serum for up to 4 h. (A) radio-HPLC injection of the labeling solution. (B) radio-HPLC injection of solution after 4 h incubation in human serum.

Stability of [^{18}F]AlF-3p-C-NETA-ePSMA-16

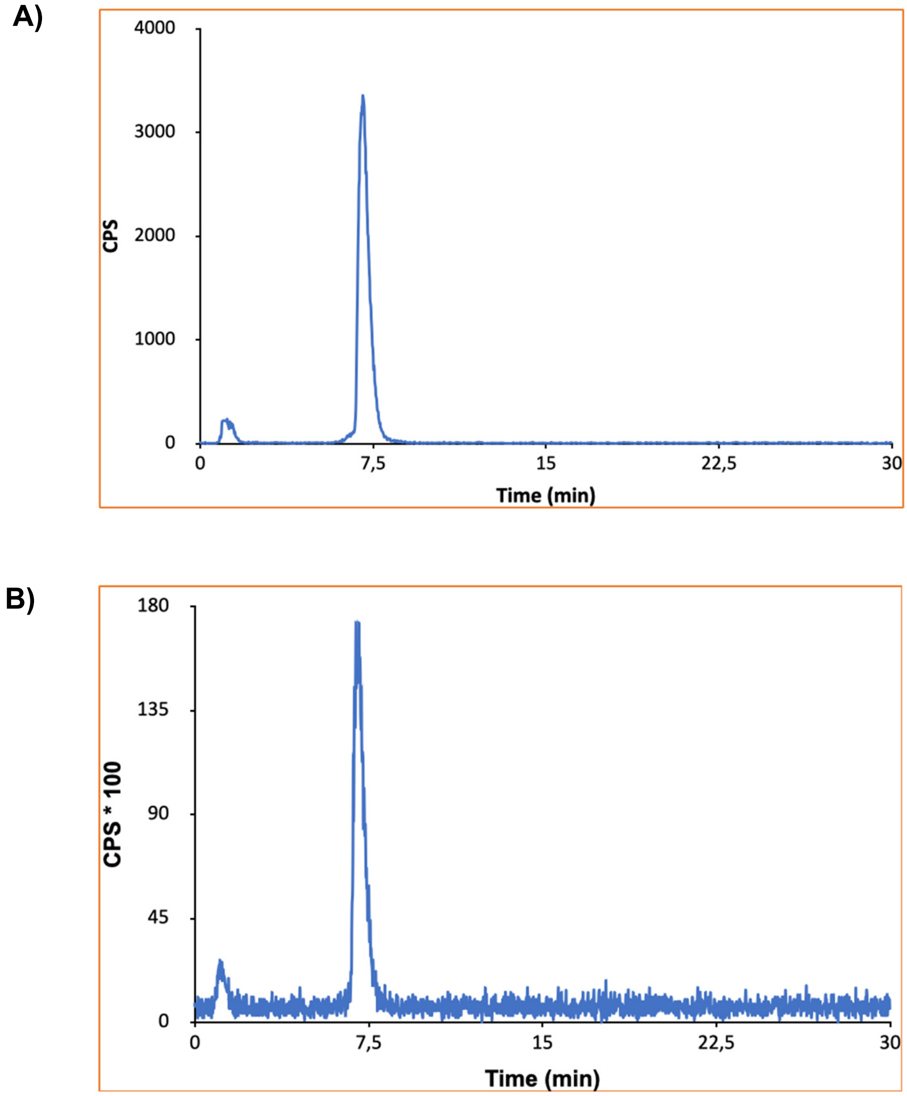

Figure S9. Stability of [^{18}F]AlF-3p-*C*-NETA-ePSMA-16 in human serum for up to 4 h. (**A**) radio-HPLC injection of the labeling solution. (**B**) radio-HPLC injection of solution after 4 h incubation in human serum.

Ex vivo biodistribution of [^{111}In]In-3p-C-NETA-ePSMA-16

Table S1: *Ex vivo* biodistribution data of [^{111}In]In-3p-*C*-NETA-ePSMA-16 (20 MBq/1 nmol) at 1, 4 and 24 h post- injection (n = 4 mice/group). A block group was also performed at 4 h. Data is represented as percentage of injected dose per gram of tissue (% ID/g).

Organ	1h	4h	4h block	24h
Blood	0.26 ± 0.19	0.01 ± 0.01	0.01 ± 0.00	0.00 ± 0.00
Tumor	1.62 ± 0.55	0.89 ± 0.58	0.12 ± 0.04	0.12 ± 0.02
Heart	0.15 ± 0.05	0.02 ± 0.00	0.02 ± 0.00	0.01 ± 0.00
Lung	0.45 ± 0.10	0.06 ± 0.01	0.05 ± 0.01	0.02 ± 0.00
Liver	0.20 ± 0.03	0.07 ± 0.01	0.07 ± 0.01	0.03 ± 0.00
Spleen	1.28 ± 0.49	0.12 ± 0.01	0.05 ± 0.01	0.03 ± 0.00
Stomach	0.31 ± 0.21	0.05 ± 0.02	0.15 ± 0.10	0.01 ± 0.00
Intestines	0.59 ± 0.13	0.60 ± 0.28	0.68 ± 0.02	0.04 ± 0.02
Pancreas	0.23 ± 0.05	0.03 ± 0.00	0.02 ± 0.01	0.01 ± 0.00
Kidney	26.70 ± 2.12	11.62 ± 0.62	9.53 ± 1.66	4.27 ± 0.87
Muscle	0.13 ± 0.09	0.04 ± 0.00	0.02 ± 0.01	0.01 ± 0.00
Skin	0.46 ± 0.15	0.24 ± 0.15	0.13 ± 0.02	0.10 ± 0.06
Bone	0.20 ± 0.05	0.03 ± 0.00	0.03 ± 0.01	0.02 ± 0.00
Prostate	1.08 ± 0.41	0.12 ± 0.07	0.07 ± 0.02	0.04 ± 0.01

In vivo biodistribution of [^{18}F]AlF-3p-C-NETA-ePSMA-16

Table S2: *In vivo* biodistribution data of [^{18}F]AlF-3p-*C*-NETA-ePSMA-16 (1.5-3.0 MBq/mouse) up to 85 minutes post injection (n = 4 mice/group). The uptake was quantified in the tumor (PC3-Pip and PC3-Flu), kidneys and liver. Data is represented as SUV_{max}.

Time (min)	Tumor (PC3-Pip)	Tumor (PC3-Flu)	Kidney	Liver
0.125	0	0	0	0
0.375	0.31 ± 0.01	0.59 ± 0.15	0.01 ± 0.06	0.31 ± 0.01
0.625	0.35 ± 0.05	1.32 ± 0.16	0.14 ± 0.04	1.58 ± 0.04
0.875	0.50 ± 0.04	1.61 ± 0.21	0.50 ± 0.01	1.71 ± 0.03
1.5	0.58 ± 0.09	1.62 ± 0.11	1.86 ± 0.06	2.02 ± 0.07
2.5	0.58 ± 0.05	1.11 ± 0.09	4.10 ± 0.08	1.36 ± 0.04
3.5	0.59 ± 0.13	0.88 ± 0.09	6.34 ± 0.02	1.16 ± 0.21
4.5	0.65 ± 0.06	0.83 ± 0.05	7.36 ± 0.02	0.98 ± 0.07
6.5	0.66 ± 0.06	0.49 ± 0.09	7.81 ± 0.01	0.59 ± 0.04
9.5	0.76 ± 0.10	0.43 ± 0.07	7.66 ± 0.02	0.45 ± 0.21
12.5	0.77 ± 0.05	0.33 ± 0.04	8.22 ± 0.02	0.32 ± 0.07
15.5	0.79 ± 0.05	0.29 ± 0.04	8.55 ± 0.05	0.31 ± 0.05
18.5	0.84 ± 0.05	0.30 ± 0.04	9.51 ± 0.02	0.3 ± 0.05
22.5	0.86 ± 0.07	0.19 ± 0.04	9.52 ± 0.05	0.19 ± 0.10
27.5	0.86 ± 0.06	0.17 ± 0.04	9.61 ± 0.08	0.18 ± 0.08
32.5	0.87 ± 0.04	0.17 ± 0.04	9.61 ± 0.04	0.18 ± 0.06
37.5	0.87 ± 0.05	0.14 ± 0.04	10.21 ± 0.08	0.17 ± 0.06
42.5	0.87 ± 0.07	0.12 ± 0.04	10.42 ± 0.04	0.17 ± 0.10
47.5	0.88 ± 0.07	0.13 ± 0.05	10.42 ± 0.04	0.17 ± 0.06
52.5	0.89 ± 0.06	0.13 ± 0.05	10.53 ± 0.05	0.16 ± 0.08
57.5	0.90 ± 0.07	0.12 ± 0.05	10.53 ± 0.05	0.16 ± 0.06
65	0.93 ± 0.04	0.11 ± 0.04	10.63 ± 0.06	0.16 ± 0.06
75	0.94 ± 0.05	0.11 ± 0.05	10.65 ± 0.08	0.16 ± 0.04
85	0.95 ± 0.05	0.09 ± 0.04	10.65 ± 0.04	0.16 ± 0.06
